# Causal effects of Epstein–Barr virus antibodies on autoimmune neuroinflammatory diseases: A generalised summary data-based Mendelian randomisation study

**DOI:** 10.1097/MD.0000000000048264

**Published:** 2026-04-10

**Authors:** Shujun Sun, Yiyong Wen, Pan Tang, Fan Xie, Tao Ding, Jun Wen, Anding Xu

**Affiliations:** a Department of Neurology and Stroke Center, The First Affiliated Hospital of Jinan University, Guangzhou, Guangdong, China; b Department of Neurology, Changde Hospital, Xiangya School of Medicine, Central South University (The First People’s Hospital of Changde City), Changde City, Hunan, China; c General Medicine, Changde Hospital, Xiangya School of Medicine, Central South University (The First People’s Hospital of Changde City), Changde City, Hunan, China.

**Keywords:** Epstein–Barr virus, genetic predisposition, multiple sclerosis, neuromyelitis optica, UK Biobank

## Abstract

Observational studies associate Epstein–Barr virus (EBV) with multiple sclerosis (MS), but causality across autoimmune neuroinflammatory diseases (ANDs) remains uncertain. This study aimed to assess the causal effects of 5 EBV antibodies on ANDs using Mendelian randomization (MR). Bidirectional, two-sample MR was performed using generalized summary-data-based MR (GSMR). Genetic instruments for EBV antibodies (anti-EBNA-1, VCA p18, ZEBRA, EA-D, IgG) were sourced from UK Biobank (UKB). Outcome data for ANDs (multiple sclerosis [MS], neuromyelitis optica [NMO], myasthenia gravis [MG], Guillain–Barré syndrome [GBS], and chronic inflammatory demyelinating polyneuropathy [CIDP]) were from FinnGen (discovery), UKB, and International Multiple Sclerosis Genetics Consortium (replication). Applying an evidence-tiered interpretation, we found a high-confidence, protective causal effect of increased ZEBRA antibody levels on MS risk (odds ratio [OR] = 0.705, *P* = 2.967 × 10^−6^), which was robust to multiple testing nominally supported in an independent cohort (IMSGC). In contrast, the association between EBNA-1 antibodies and increased MS risk (OR = 1.284, *P *= 2.450 × 10^−4^) was graded as suggestive as it showed nominal support only in one of 2 validation cohorts (UKB) and was substantially attenuated after excluding HLA-region single nucleotide polymorphisms, indicating shared genetic architecture rather than direct causation. Steiger directionality tests confirmed the primary causal direction was from antibodies to MS. Results for NMO, MG, GBS, and CIDP were inconclusive due to limited statistical power, and thus no definitive conclusions can be drawn regarding EBV antibodies and these diseases. This study provides high-confidence genetic evidence for a protective role of ZEBRA antibodies in MS, which was robust to multiple testing and nominally supported in an independent cohort [International MS Genetics Consortium (IMSGC)], and suggestive evidence for an HLA-mediated link between EBV-encoded nuclear antigen-1 (EBNA-1) and MS which showed nominal support only in one of 2 validation cohorts. The role of EBV in other ANDs warrants investigation in larger, well-powered cohorts.

## 1. Introduction

Autoimmune neuroinflammatory diseases (ANDs) are a category of disorders wherein the immune system aberrantly targets and attacks the nervous system, resulting in structural and functional damage.^[1,2]^ ANDs primarily include multiple sclerosis (MS), neuromyelitis optica (NMO), Guillain–Barré syndrome (GBS), chronic inflammatory demyelinating polyneuropathy (CIDP), and myasthenia gravis (MG). MS and NMO affect the central nervous system (CNS), GBS and CIDP affect the peripheral nervous system, and MG affects the neuromuscular junction. These conditions cause neuronal or axonal damage, demyelination, nerve-muscle junction damage, and other pathological changes.^[3,4]^ Genetic and environmental factors play a role in their development.^[5]^ Infections are also a common trigger for autoimmune diseases.^[6]^

Epstein–Barr virus (EBV), a herpesvirus that infects over 90% of the world’s population, has been implicated as an important risk factor for MS.^[7]^ MS risk increases 32-fold following EBV infection.^[8]^ Moreover, geometric mean titers of antibodies against the Epstein–Barr nuclear antigen (EBNA) complex and its specific component, EBV-encoded nuclear antigen-1 (EBNA-1), are significantly higher in MS cases than in matched controls.^[9]^ Seropositivity for serum immunoglobulin (IgG) antibodies to the EBV early antigen (EA) is also significantly higher in patients with NMO spectrum disorder (NMOSD) than in healthy controls (HCs). In contrast, the serum levels of anti-EBNA-1 IgG are significantly lower in patients with NMOSD than in HCs.^[10]^ Serum antibody titers against EBV viral capsid antigen (EBV-VCA) are also higher in patients with GBS than in HCs.^[11]^ Patients with CIDP are consistently seropositive for EBV-specific IgG, and the disease is associated with moderately elevated IgG responses to EBV antigens expressed during B-cell transformation and active viral replication. In addition, cellular EBV copy number is 3 times higher in patients with CIDP than in controls.^[12]^ However, the association between EBV infection and MG pathogenesis remains unclear.^[13,14]^

Mendelian randomization (MR) is a novel approach that may effectively mitigates previous limitations. Using genetic variants as instrumental variables (IVs), MR reduces the confounding effects of environmental factors and avoids reverse causation bias.^[15,16]^ Generalised summary-data-based MR (GSMR) offers distinct advantages over conventional MR as it accounts for linkage disequilibrium (LD) among genetic variants, improving analytical efficiency. Additionally, GSMR employs the heterogeneity in dependent instruments (HEIDI) test to identify outlier instruments and exclude pleiotropic single nucleotide polymorphisms (SNPs).^[17]^

Although previous studies have suggested an association between EBV-specific antibodies and ANDs, whether this relationship is causal or EBV-specific antibodies serve merely as biomarkers in ANDs remains unclear. Hence, this study aimed to analyze causal relationships between antibody-mediated immune responses to EBV infection and ANDs. Given that previous epidemiological studies have not definitively established a causal link between EBV infection and ANDs, we used large-scale genome-wide association study (GWAS) genotypic and phenotypic data to perform bidirectional two-sample MRs and GSMRs.

## 2. Methods

### 2.1. Ethics approval and informed consent

This study was approved by the Ethics Committee of Changde First People’s Hospital (Approval No.: 2025-505-01). Informed consent was obtained from all participants prior to enrollment, and ethical approval was obtained for each cohort. Publicly available datasets were used for all data in our study. Informed consent was not required.

### 2.2. Study design and data sources

This study was conducted in 2 phases. First, we investigated whether EBV infection, characterized by 5 anti-EBV antibody traits, had a causal effect on several ANDs, namely, MS, NMO, MG, GBS, and CIDP. Subsequently, using summary-level data from the FinnGen database and the UK Biobank (UKB), reverse causality was explored, specifically whether ANDs influenced EBV infection. All genetic data used in this study were derived from individuals of European ancestry. This focus enhanced genetic homogeneity and reduced population stratification bias but limited the generalisability of our findings to other ancestral populations.

The study included Europeans. Summary GWAS data for the 5 EBV antibodies were sourced from the MRC-IEU UKB OpenGWAS Project database (https://gwas.mrcieu.ac.uk/). The antibodies (and their properties) assessed were anti-EBV IgG seropositivity (GWAS ID: ebi-a-GCST90006897, n = 8735), EBV early antigen-D (EA-D) antibody levels (GWAS ID: ebi-a-GCST90006898, n = 7763), EBV nuclear antigen-1 (EBNA-1) antibody levels (GWAS ID: ebi-a-GCST90006899, n = 7972), EBV viral capsid antigen p18 (EBV-VCA p18) antibody levels (GWAS ID: ebi-a-GCST90006900, n = 8518), and EBV transcriptional transactivator protein Z EBV replication activator (ZEBRA) antibody levels (GWAS ID: ebi-a-GCST90006901, n = 8191). Antibody levels were expressed as the logarithmic transformation of median fluorescence intensity.^[18]^

IVs for MS, MG, and GBS were sourced from the latest FinnGen database (https://r11.finngen.fi/), with respective sample sizes of 452,390 (comprising 2620 cases and 449,770 controls for MS), 450,074 (504 cases and 449,570 controls for MG), and 446,354 (489 cases and 445,865 controls for GBS), respectively. All cases were classified according to the International Classification of Diseases, 10th Revision. To validate the significant findings, genome-wide summary data for MS from the International Multiple Sclerosis Genetics Consortium (IMSGC), which included 47,429 cases and 68,374 controls,^[19]^ were used. Similarly, summary data from the UKB dataset (https://gwas.mrcieu.ac.uk/datasets/ukb-b-17670/), which comprised 1679 cases and 461,254 controls, were obtained.

NMO-related GWAS data were obtained from the largest and most recent NMO GWAS meta-analysis, which included 215 NMO cases and 1244 controls; the primary study involved the 2006 NMO diagnostic criteria.^[20]^ Genetic association data for CIDP were derived from the UKB dataset, which included 456,348 Europeans (425 cases and 455,723 controls). The associations were established using a fast genome-wide association generalized linear mixed model and were obtained from the relevant catalogue.^[21]^

All antibody phenotypes (log-MFI) were standardized to a mean of 0 and SD of 1 in the original serology GWAS.^[18]^ Thus, all MR estimates in this study represented the effect per 1 SD increase in standardized log-MFI antibody levels. To quantify potential sample overlap, we compared the total sample sizes of the exposure and outcome data sources. Given that the UKB sample size for the antibody GWAS (n = 7763–8735) is much smaller than that of the UKB outcome GWAS (>460,000), we calculated a conservative upper bound for the maximum possible overlap by assuming all antibody GWAS participants were also included in the outcome GWAS. This yielded an overlap of <2% (7735/ 460,000 ≈ 1.7%). Sensitivity analyses using the IMSGC MS dataset (which included no UKB participants) confirmed that overlap did not materially change the results.

### 2.3. IV selection

To facilitate robust GSMRs, a significance threshold of *P* < 5 × 10^−6^ for anti-EBV antibodies and autoantibody (AND) IVs was adopted, as recommended by Wang et al.^[22]^ This threshold was selected because setting the significance level at *P* < 5 × 10^−8^ or *P* < 5 × 10^−7^ would result in fewer than 2 IVs for some characteristics. SNPs with minor allele frequencies of <0.01 were excluded. LD among SNPs was resolved via clustering, with an *r*^2^ threshold of <0.001 within a 10 Mb clustering distance. Palindromic SNPs were also removed. Subsequently, exposure and outcome data were harmonized. We then applied the Steiger directionality test to the harmonized IVs to validate the presumed causal direction.

The proportion of variance explained by the IVs is calculated using the formula:


$$\begin{array}{l} R^{2}=\frac{2\times \beta^{2}\times EAF\times \left(1-EAF\right)}{\begin{array}{lll} & 2\times \beta^{2}\times EAF\times \left(1-EAF\right)\; & +2\times \beta^{2}\times N\times EAF\times \left(1-EAF\right)\; \end{array}}, \end{array}$$

where *N* represents the sample size^[23]^.

The strength of the genetic instrument was assessed using the *F*-statistic, calculated as follows:


$$\begin{array}{l} F=\frac{x^{2}\times \left(N-2\right)}{\left(1-R^{2}\right)}. \end{array}$$

An *F*-statistic > 10 indicated a strong instrument; IVs with *F*-statistics < 10 were deemed weak and excluded from the analysis. To ensure that the selected SNPs were not associated with other relevant confounders, such as smoking, body mass index, physical activity, or serum 25-hydroxyvitamin D levels, the Single Nucleotide Polymorphism Annotator and Browser (SNiPA) (http://www.snipa.org/) was used.

Furthermore, to ensure the robustness of our genetic instruments, a conditional/independent SNP selection was implemented using GCTA-COJO to mitigate redundancy due to LD.

### 2.4. Sensitivity analyses and robustness checks

To address potential concerns regarding IV validity and causal direction, we conducted complementary sensitivity analyses. Firstly, Steiger filtering was applied to verify the presumed direction of causality for each SNP, ensuring that the genetic instruments explained more variance in the exposure than in the outcome. Secondly, to evaluate the robustness of our primary causal estimates, we re-ran the GSMR analysis using IVs derived from the enhanced selection pipeline (i.e. after applying GCTA-COJO and Steiger filtering). Lastly, the HEIDI outlier method and the MR-PRESSO global test was systematically employed to identify and account for potential horizontal pleiotropy.

Furthermore, although our primary analyses relied on LD estimates from the 1000 Genomes European reference panel due to computational and data availability constraints, we performed extensive sensitivity analyses (including conditional IV selection, Steiger filtering, MR-RAPS, leave-one-out, and cohort-leave-out analyses) to ensure the robustness of our findings against potential variations in LD structure. The consistency of results across these methods suggests that our core inferences are not substantially influenced by the choice of LD reference panel. Detailed genetic instrument data from the conditional selection and Steiger filtering pipeline are available in additional supplemental tables.”

### 2.5. Mendelian randomisation

#### 2.5.1. GSMR:

GSMR was used in the primary analyses. Briefly, this method involves using multiple, nearly independent IVs to conduct MRs, testing for a potential causal relationship between a risk factor (or phenoty ent cohorts.^[24]^ For genetic instruments related to anti-EBV antibodies, the clumping algorithm was used to select genome-wide significant SNPs for each trait. This process involved applying an *r*^2^ threshold of 0.05 and a *P*-value threshold of 5 × 10^−6^. LD was estimated using the 1000 Genomes (1000G) Phase 3 European samples as the reference population. In the reverse GSMR, independent SNPs were selected from the GWAS summary statistics of autoantibodies (ANDs) using an LD threshold of *r*^2^ < .05 and a *P*-value threshold of 5 × 10^−6^. Subsequently, the HEIDI outlier method was applied to exclude instruments with potential pleiotropic effects, using a *P*-value threshold of .01 for filtering.^[24]^

#### 2.5.2. Univariable two-sample MR:

Univariable two-sample MR (TSMR) was used to estimate the total causal effects of EBV antibodies on ANDs. Five MR methods were applied, namely inverse variance weighting (IVW), MR Egger, weighted median, simple mode, and weighted mode. The IVW method was used as the primary approach for interpreting results.^[25]^ Reverse MRs were also conducted to investigate potential reverse causality between ANDs and EBV antibodies. Bonferroni correction was applied to account for multiple comparisons and mitigate the increased risk of false positives. We pre-specified a single hypothesis family encompassing all bidirectional MR analyses, resulting in 50 independent tests (n = 50). The study-wide significance threshold was set at α = 0.05/50 = 1.0 × 10^−3^.^[26]^ This threshold was applied consistently to all primary GSMR and TSMR estimates. Based on this, associations were categorized as follows: “significant after Bonferroni correction” (*P* < 1.0 × 10^−3^), “suggestive” (1.0 × 10^−3^ ≤ *P* < .05), or “not significant” (*P* ≥ .05).

The MR Egger intercept and MR-Pleiotropy RESidual Sum and Outlier (PRESSO) tests were used to detect pleiotropy. A *P*-value of ≤.05 for either test suggested the presence of pleiotropic IVs. To address this, we iteratively performed MR Egger intercept and MR-PRESSO tests, removing any detected outliers until no evidence of horizontal pleiotropy remained. Additionally, scatter and forest plots were visually inspected to assess the “no horizontal pleiotropy” assumption of MR. Similarly, Cochran *Q* statistic was used to evaluate heterogeneity among IVs, with a *P*-value of >.05 indicating no significant heterogeneity.

To assess the robustness of the identified causal associations, 2 sensitivity analyses were conducted, namely the MR Egger intercept test and the leave-one-out analysis. The intercept from the MR Egger test was used to estimate the degree of directional pleiotropy, while the leave-one-out analysis was adopted to determine whether significant results were driven by any single SNP. The statistical power for our MR analyses was assessed using the method for binary outcomes described by Burgess.^[27]^ All statistical analyses were performed using R version 4.4.1, with packages including TwoSampleMR, MRPRESSO, and GSMR2.

## 3. Results

### 3.1. Study overview

Detailed information on EBV antibodies and ANDs is presented in Table 1. First, the presence of positive causal effects of EBV-related antibodies on ANDs was examined using forward GSMR and TSMR. Subsequently, we assessed whether ANDs had a reverse causal effect on EBV antibodies using reverse GSMR and TSMR. To establish robust causal inference, a comprehensive replication strategy was implemented across independent datasets. EBNA-1→MS associations were validated using both UKB and IMSGC datasets. Similarly, for ZEBRA→MS relationships, we conducted replication analyses in these independent cohorts to distinguish consistently validated effects from dataset-specific findings.

**Table 1:** Baseline characteristics of participants.

Exposure or outcome	Data source	Phenotype	Year	Sample sizes	Ancestry
Antibody immune responses to EBV	Butler-Laporte G et al^[18]^	Anti-EBV IgG seropositivity	2020	8735	European
		EBV EA-D antibody levels	2020	7763	European
		EBV EBNA-1 antibody levels	2020	7972	European
		EBV VCA p18 antibody levels	2020	8518	European
		EBV ZEBRA antibody levels	2020	8191	European
ANDs	FinnGen	MS	2024	2620 cases and 449,770 controls	European
	IMSGC	MS	2019	47,429 cases and 68,374 controls	European
	UKB	MS	2018	1679 cases and 461,254 controls	European
	Estrada K et al^[20]^	NMO	2018	215 cases and 1244 controls	European
	FinnGen	MG	2024	504 cases and 449,570 controls	European
	FinnGen	GBS	2024	489 cases and 445,865 controls	European
	Jiang L et al^[21]^	CIDP	2021	425 case and 455,723 controls	European

ANDs = autoimmune neuroinflammatory diseases, CIDP = chronic inflammatory demyelinating polyneuropathy, EA-D = early antigen-D, EBNA = Epstein–Barr virus nuclear antigen 1, EBV = Epstein–Barr virus, GBS = Guillain–Barré syndrome, IgG = immunoglobulin gamma, IMSGC = International Multiple Sclerosis Genetics Consortium, MG = myasthenia gravis, MS = multiple sclerosis, NMO = neuromyelitis optica, UKB = UK Biobank, VCA = viral capsid antigen, ZEBRA = Z EBV replication activator.

### 3.2. Selected IVs

Specific information on the IVs for EBV antibodies is presented in Tables S1–S3 (Supplemental Digital Content, https://links.lww.com/MD/R626) while IVs for ANDs are detailed in Tables S4–S6 (Supplemental Digital Content, https://links.lww.com/MD/R626). All IVs had *F*-statistics > 10, confirming the exclusion of weak instruments. Additionally, all IVs were confirmed to be unrelated to the potential confounders, including smoking, body mass index, physical activity, and serum 25-hydroxyvitamin D levels. Complete SNP-level data for the IVs used in both the primary and robustness analyses are provided in additional supplemental tables.

### 3.3. Causal effect of EBV-specific antibodies on AND risk

Effect estimates are presented as odds ratios per 1 SD increase in antibody levels, where all antibody traits were standardized log-MFI values in the original GWAS. The primary GSMR and TSMR results for the causal effects of EBV-associated antibodies on ANDs are presented in Table 2. After applying the pre-specified Bonferroni correction (*P* < 1.0 × 10^−3^), The GSMR statistics revealed a significant causal effect of EBV EBNA-1 and EBV ZEBRA antibody levels on MS. A 1 SD increase in EBV EBNA-1 antibody levels was associated with an increased MS risk (odds ratio [OR]: 1.284, 95% confidence interval [CI]: 1.123–1.467, *P* = 2.450 × 10^−4^). The 3 primary TSMR statistical methods yielded consistent results (Table 2). The causal relationship between EBV EBNA-1 antibody levels and MS was nominally significant in the UKB dataset (*P* < .05) but not in the IMSGC cohort (Table 3), indicating an asymmetric validation pattern across cohorts. This asymmetric replication may reflect differences in phenotype definitions. The IMSGC dataset represents a specialized multiple sclerosis genetics consortium with potentially more stringent case ascertainment. Meanwhile, the UKB employs broader epidemiological criteria. To further probe the robustness of the EBNA-1→MS association, we conducted a cohort-leave-out sensitivity analysis by systematically excluding IVs that showed significant associations with MS (see Table S7, Supplemental Digital Content, https://links.lww.com/MD/R627). Notably, when the 2 strongest instrumental SNPs – rs6927022 and rs9273857, both located in the HLA region and known to be pivotal in EBV immune response and MS susceptibility – were removed, the causal effect estimate was substantially attenuated and lost statistical significance (GSMR: OR = 0.927, *P* = .422). This finding suggests that the observed association between EBNA-1 antibodies and MS risk is predominantly driven by a shared genetic background in the HLA region, rather than a direct effect of the antibody itself.

**Table 2:** Main MR results considering antibodies against EBV as exposure.

Exposure	Outcome	GSMR (main analysis)		MR Egger		Weighted median		Inverse variance weighted	
		OR (95% CI)	*P* *	OR (95% CI)	*P* *	OR (95% CI)	*P* *	OR (95% CI)	*P* *
Anti-EBV IgG seropositivity	NMO	1.517 (1.007–2.287)	.046	0.285 (0.006–13.094)	.636	1.671 (1.026–2.721)	.039	1.533 (1.032–2.276)	.034
EBV EBNA-1 antibody levels	MS	1.284 (1.123–1.467)	2.450 × 10^−4^	1.849 (1.355–2.523)	2.577 × 10^−3^	1.657 (1.356–2.023)	7.528 × 10^−7^	1.462 (1.221–1.750)	3.608 × 10^−5^
EBV VCA p18 antibody levels	MS	1.026 (0.859–1.226)	.777	1.630 (1.080–2.462)	.049	1.306 (0.979–1.743)	.070	1.261 (1.017–1.564)	.034
EBV ZEBRA antibody levels	MS	0.705 (0.609–0.817)	2.967 × 10^−6^	0.600 (0.404–0.892)	.035	0.619 (0.510–0.751)	1.086 × 10^−6^	0.654 (0.532–0.804)	5.725 × 10^−5^
	NMO	0.387 (0.154–0.971)	.043	0.252 (0.028–2.236)	.262	0.405 (0.129–1.278)	.123	0.376 (0.153–0.927)	.034

CI = confidence interval, EBNA = Epstein–Barr virus nuclear antigen 1, EBV = Epstein–Barr virus, GSMR = generalized summary-data-based Mendelian randomization, IgG = immunoglobulin gamma, MR = Mendelian randomization, NMO = neuromyelitis optica, OR = odds ratio, VCA = viral capsid antigen, ZEBRA = Z EBV replication activator.

* Raw *P*-values are reported. A Bonferroni-adjusted significance threshold of *P* < 1.0 × 10^−3^ was applied to the entire hypothesis family. All causal inferences from GSMR tests are definitively interpreted as follows: significant after Bonferroni correction (*P* < 1.0 × 10^−3^), suggestive (*P* ≥ 1.0 × 10^−3^ and <.05), or not significant (*P* ≥ .05). ORs represent the effect per 1 SD increase in standardized log-MFI antibody level.

**Table 3:** Validation of causal associations of EBV EBNA-1 and ZEBRA antibody levels with MS.

Exposure*	Outcome	Inverse variance weighted		Pleiotropy	
		OR (95% CI)	*P*	Egger intercept	*P*
EBV EBNA-1 antibody levels	MS (IMSGC)	0.561 (0.166–1.896)	.352	/†	/†
EBV ZEBRA antibody levels		0.176 (0.037–0.844)	.03	0.463	0.2
EBV EBNA-1 antibody levels	MS (UKB)	1.003 (1.002–1.005)	2.882 × 10^−5^	−8.964 × 10^−5^	.815
EBV ZEBRA antibody levels		0.997 (0.993–1.000)	.083	/†	/†

CI = confidence interval, EBNA-1 = Epstein–Barr virus nuclear antigen 1, EBV = Epstein–Barr virus, IMSGC = International Multiple Sclerosis Genetics Consortium, IVs = instrumental variables, MS = multiple sclerosis, OR = odds ratio, UKB = UK Biobank, ZEBRA = Z EBV replication activator.

* In the validation phase, the *P*-value for screening IVs was set at <5 × 10^−8^, differing from the *P*-value criterion in Table 2. The significant causal associations for EBNA-1 and ZEBRA with MS identified in the primary analysis (Table 2) were validated using independent GWAS summary statistics from 2 different sources (UK Biobank and IMSGC). A nominal threshold of *P* < .05 was used to confirm the consistency of these associations in the validation cohorts.

† Too few instrumental variables to test for pleiotropy. ORs represent the effect per 1 SD increase in standardized log-MFI antibody level.

A 1 SD increase in EBV ZEBRA antibody levels was associated with a decreased MS risk (OR: 0.705, 95% CI: 0.609–0.817, *P* = 2.967 × 10^−6^). Consistent results were observed using the weighted median and IVW methods (Table 2). Similarly, the protective effect of EBV ZEBRA antibody levels on MS risk showed an asymmetric validation pattern. It was nominally significant in the IMSGC cohort (*P* < .05) but not in the UKB dataset (Table 3). This pattern indicates that the association was corroborated in one independent cohort but not consistently across all validation datasets. The differential replication may be attributed to variations in instrument sets and exposure scaling between cohorts, as the genetic instruments were selected using cohort-specific LD reference panels and significance thresholds.

Robustness analysis using stringent IV selection (GCTA-COJO and Steiger filtering) strengthened the key causal inferences for EBV EBNA-1 (OR = 1.807, *P* = 4.176 × 10^−17^) and ZEBRA antibodies (OR = 0.598, *P* = 9.676 × 10^−9^) on MS, confirming the primary results (see Table S8, Supplemental Digital Content, https://links.lww.com/MD/R627; see Fig. S1, Supplemental Digital Content, https://links.lww.com/MD/R628). A comprehensive assessment of IV robustness is detailed in Table S9 (Supplemental Digital Content, https://links.lww.com/MD/R627). Following conditional and Steiger filtering, all exposure-outcome pairs were instrumented by a sufficient number of strong variants (all minimum *F*-statistics > 21.2, median *F*-statistics range: 28.5–69.4). The Steiger filtering success rate was high (majority 100%), providing strong support for the presumed direction of causality from EBV antibodies to ANDs. Furthermore, MR-PRESSO global tests indicated no significant evidence of horizontal pleiotropy (all *P*-values > .05).

Although the 3 primary TSMR statistical methods suggested a potential adverse causal effect of EBV-VCA p18 antibody levels on MS, this finding was not confirmed by GSMR and was not significant after multiple testing correction.

The GSMR results also identified suggestive, but not statistically significant after correction, potential causal effects of anti-EBV IgG seropositivity and EBV ZEBRA antibody levels on NMO. Anti-EBV IgG seropositivity may increase NMO risk (OR: 1.517, 95% CI: 1.007–2.287, *P* = .046), with the weighted median and IVW methods yielding similar results (Table 2). Conversely, a 1 SD increase in EBV ZEBRA antibody levels was associated with a decreased NMO risk (OR: 0.387, 95% CI: 0.154 to 0.971, *P* = .043), as shown by the IVW analysis (Table 2). However, power analysis indicated limited ability to detect moderate effects for NMO (see Table S10, Supplemental Digital Content, https://links.lww.com/MD/R627), and thus, these findings require validation in larger cohorts.

**Figure 1.**
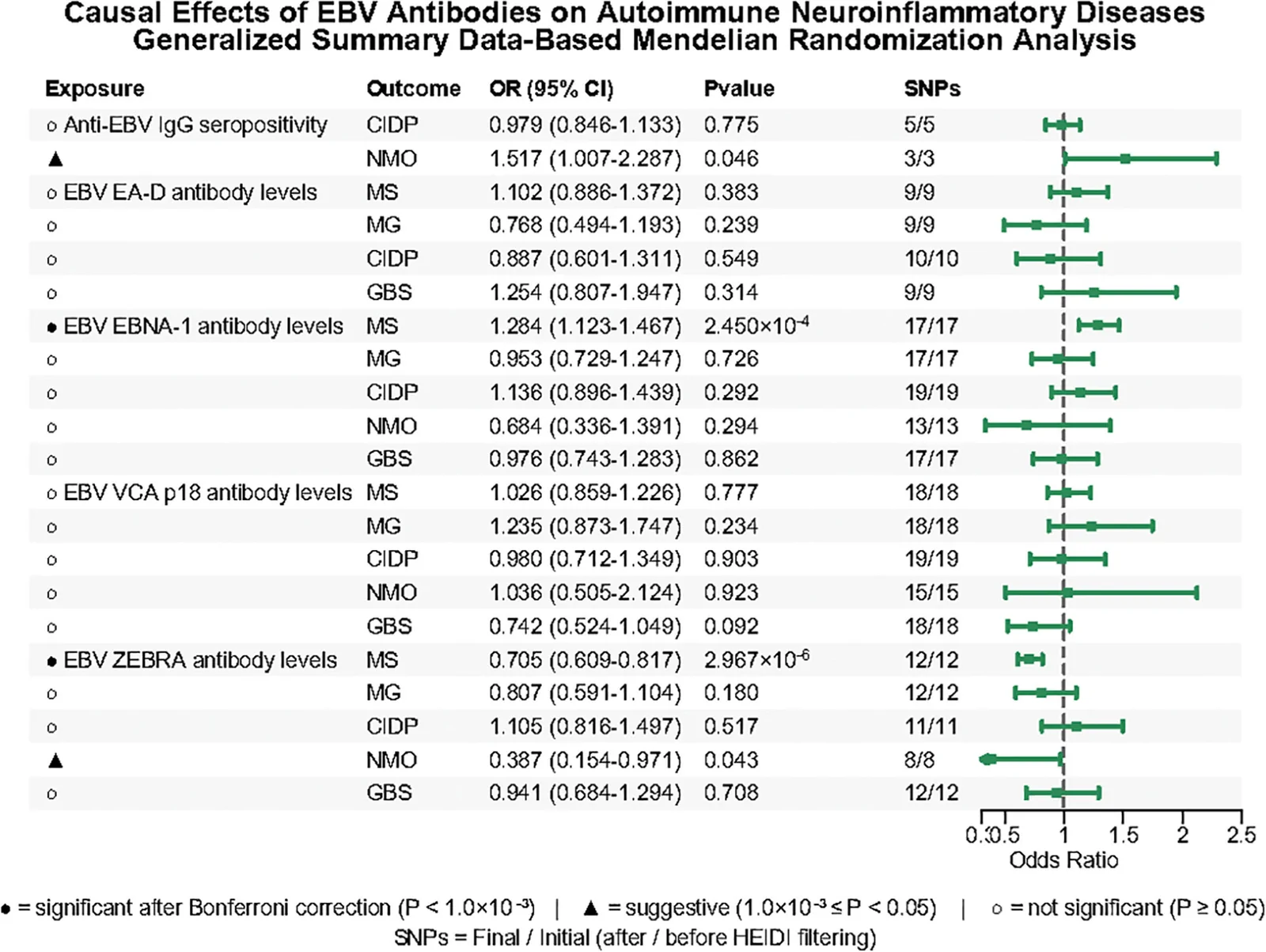
Forest plot displaying the generalized summary-data-based Mendelian randomization (GSMR) results for the effect of Epstein–Barr virus (EBV) antibodies on autoimmune neuroinflammatory diseases (ANDs). The analysis is conducted using GSMR, with a Bonferroni-corrected significance threshold set at *P* = 1.0 × 10^–3^. ANDs = autoimmune neuroinflammatory diseases, EBV = Epstein–Barr virus, GSMR= generalised summary-data-based Mendelian randomization.

GSMR revealed no causal effects of EBV antibody immune response profiles on MG, GBS, or CIDP that were significant after Bonferroni correction (Fig. 1). We evaluated the statistical power of these negative findings. As detailed in Table S10 (Supplemental Digital Content, https://links.lww.com/MD/R627), our GSMR analyses for the non-MS ANDs (NMO, MG, GBS, and CIDP) were severely underpowered, with the ability to detect only very large (for NMO) or extreme, often biologically implausible, effect sizes (for MG, GBS, and CIDP). In contrast, the analyses for MS were adequately powered. Therefore, the null associations observed for non-MS ANDs should be interpreted as inconclusive due to limited power, rather than as evidence of no causal effect.

When performing GSMR, the IVs for anti-EBV IgG seropositivity in MS, MG, and GBS, as well as EBV EA-D antibody levels in NMO, were deemed insufficient for GSMR (Fig. 1). Therefore, the causality for these associations was based on the TSMR results, which revealed no significant effects after multiple testing correction (see Fig. S2, Supplemental Digital Content, https://links.lww.com/MD/R628). Heterogeneity was observed among the IVs of EBV EBNA-1 (IVW: *Q* = 21.328, *P* = .046) and EBV ZEBRA (IVW: *Q* = 18.284, *P* = .032) antibody levels in their effects on MS (see Table S11, Supplemental Digital Content, https://links.lww.com/MD/R626). For MS and NMO, all *P*-values from the MR Egger intercepts were greater than .05, indicating no evidence of horizontal pleiotropy between IVs and outcomes (Tables S11 and S12, Supplemental Digital Content, https://links.lww.com/MD/R626).

The MR-PRESSO distortion test revealed outlier SNPs, which were removed. The leave-one-out sensitivity analysis (see Figs. S3–S12, Supplemental Digital Content, https://links.lww.com/MD/R628) revealed distinct, divergent patterns for EBNA-1 and ZEBRA antibodies (see Figs. S4 and S5, Supplemental Digital Content, https://links.lww.com/MD/R628, respectively). For EBNA-1, the positive causal estimate was overwhelmingly driven by a single SNP, rs6927022, as its removal attenuated the estimate to the null. In contrast, the protective association for ZEBRA was highly robust. Although removal of the strongest instrument, that is, rs2647025 (located within the MHC/HLA region (6p21.32)), led to a widening of the confidence interval, the point estimate remained consistent in direction and magnitude with the overall analysis, indicating the stability of the findings.

As a complementary robust estimation method resistant to pleiotropy, we performed MR-RAPS analysis (see Figs. 13 and 14, Supplemental Digital Content, https://links.lww.com/MD/R628). The results corroborated the association between EBV antibodies and MS. A 1 SD increase in EBNA-1 antibody levels was associated with increased MS risk (β = 0.391, 95% CI: 0.249–0.534, *P* = 5.38 × 10^−8^). In contrast, a 1 SD increase in ZEBRA antibody levels was associated with a decreased risk (β = −0.443, 95% CI: −0.596–−0.291, *P* = 1.81 × 10^−8^). Normality tests of standardized residuals were non-significant (*P* > .05 for both), supporting the robustness of the MR-RAPS estimates (see Table S13, Supplemental Digital Content, https://links.lww.com/MD/R627).

To validate the direction of our primary causal hypothesis from EBV antibodies to MS, Steiger directionality tests were performed with the data used for the forward GSMR analysis. The results confirmed that the genetic instruments explained significantly more variance in the EBV antibodies (EBNA-1, VCA p18, ZEBRA) than in MS (all *P* < 1.0 × 10^−80^), providing strong support for the causal direction from EBV antibodies to MS (see Table S14, Supplemental Digital Content, https://links.lww.com/MD/R627).

### 3.4. Power analysis for non-MS ANDs

Given the limited number of cases for NMO, MG, GBS, and CIDP, we performed a post hoc power analysis to assess our ability to detect plausible effect sizes. As detailed in Table S10 (Supplemental Digital Content, https://links.lww.com/MD/R627), our analyses for these outcomes were severely underpowered, with minimum detectable ORs ranging from 1.8 to >5 for most antibody-disease pairs. Therefore, the null associations observed for NMO, MG, GBS, and CIDP should be interpreted as inconclusive due to insufficient statistical power, rather than as evidence of no causal effect.

### 3.5. Reverse MRs of ANDs on EBV antibodies

GSMRs were performed to explore reverse causal relationships between ANDs and EBV antibodies. The results showed a significant positive association between MS and increased EBV EBNA-1 (OR: 1.117, 95% CI: 1.082–1.152, *P* = 6.170 × 10^−12^) and EBV-VCA p18 (OR: 1.069, 95% CI: 1.037–1.102, *P* = 1.570 × 10^−5^) antibody levels after Bonferroni correction. The GSMR showed a suggestive negative association between MS and EBV ZEBRA antibody levels (OR: 0.952, 95% CI: 0.924–0.981, *P* = 1.459 × 10^−3^). However, this association did not meet the strict Bonferroni-corrected significance threshold. Conversely, TSMRs did not confirm an inverse association of MS with EBV EBNA-1, EBV-VCA p18, or EBV ZEBRA antibody levels (Table 4).

**Table 4:** Main MR results considering MS as exposure.

Exposure	Outcome	GSMR (main analysis)		MR Egger		Weighted median		Inverse variance weighted	
		OR (95% CI)	*P* *	OR (95% CI)	*P* *	OR (95% CI)	*P* *	OR (95% CI)	*P* *
MS	EBV EBNA-1 antibody levels	1.117 (1.082–1.152)	6.133 × 10^−12^	1.074 (0.965–1.194)	.199	1.008 (0.955–1.064)	.779	1.027 (0.987–1.070)	.190
	EBV VCA p18 antibody levels	1.069 (1.037–1.102)	1.570 × 10^−5^	0.996 (0.902–1.100)	.937	1.014 (0.963–1.068)	.595	0.988 (0.952–1.025)	.509
	EBV ZEBRA antibody levels	0.952 (0.924–0.981)	.001	1.023 (0.927–1.129)	.650	0.990 (0.938–1.044)	.700	0.983 (0.948–1.020)	.366

CI = confidence interval, EBNA-1 = Epstein–Barr virus nuclear antigen 1, EBV = Epstein–Barr virus, GSMR = generalized summary-data-based Mendelian randomization, MR = Mendelian randomization, MS = multiple sclerosis, OR = odds ratio, VCA = viral capsid antigen, ZEBRA = Z EBV replication activator.

* Raw *P*-values are reported. A Bonferroni-adjusted significance threshold of *P* < 1.0 × 10^−3^ was applied to the entire hypothesis family. All causal inferences from GSMR tests are definitively interpreted as follows: significant after Bonferroni correction (*P* < 1.0 × 10^−3^), suggestive (day 1.0 × 10^−3^ ≤ *P* < .05), or not significant (*P* ≥ .05). ORs represent the effect per 1 SD increase in standardized log-MFI antibody level.

**Figure 2.**
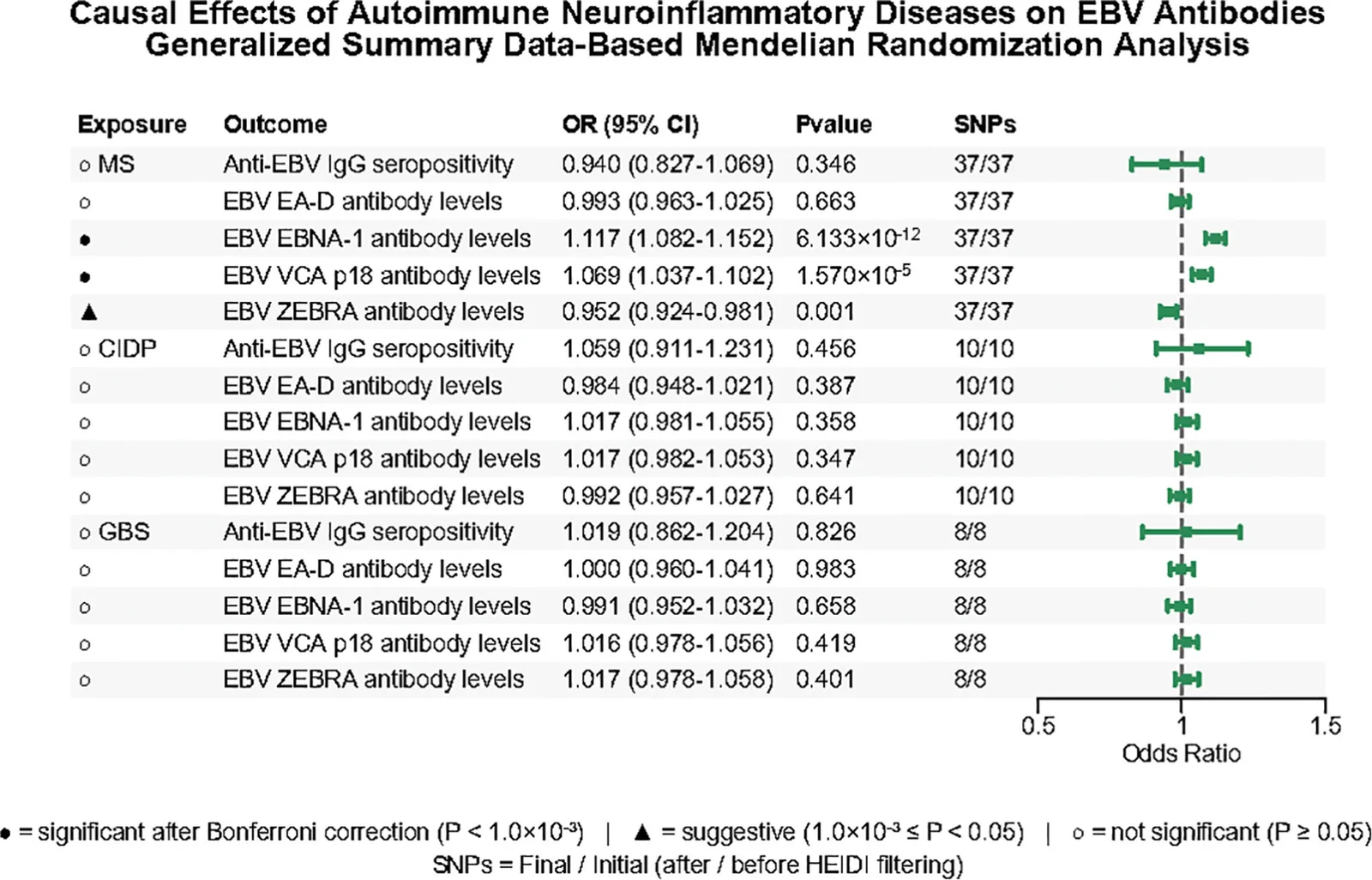
Forest plot displaying the GSMR results for the effect of ANDs on EBV antibodies. The analysis is conducted using GSMR, with a Bonferroni-corrected significance threshold of *P* = 1.0 × 10^−3^. ANDs = autoimmune neuroinflammatory diseases, EBV = Epstein–Barr virus, GSMR= generalised summary-data-based Mendelian randomization.

Reverse GSMRs did not indicate a causal effect of GBS or CIDP on EBV antibodies (Fig. 2). Additionally, TSMR did not show reverse relationships between NMO and EBV antibodies (see Table S15, Supplemental Digital Content, https://links.lww.com/MD/R626). Reverse GSMR statistics for NMO and MG against EBV antibodies could not be performed owing to the absence of effect allele frequency values in the NMO GWAS data and an insufficient number of valid IVs for MG. Therefore, the assessment of reverse causality for NMO and MG against EBV antibodies was based on the TSMR statistics, which indicated no significant reverse causality (see Fig. S15, Supplemental Digital Content, https://links.lww.com/MD/R628).

Interpretation of discordant reverse MR results: It is important to note that while reverse GSMR suggested significant associations of MS with EBNA-1 and VCA p18 antibody levels, reverse TSMR did not confirm these effects. This discrepancy likely reflects methodological differences: GSMR accounts for LD among instruments and uses HEIDI filtering, which may capture pleiotropic pathways more sensitively, whereas TSMR methods are more conservative. Crucially, Steiger directionality tests robustly supported the forward direction (antibodies→MS) and rejected the reverse path (all *P* < .01). Therefore, the significant reverse GSMR signals should not be interpreted as evidence of true reverse causality but rather as indicative of shared genetic architecture – primarily HLA-mediated pleiotropy – that influences both MS susceptibility and antibody response. This underscores the importance of triangulating multiple MR methods and directionality checks to draw reliable causal inferences.

All *P*-values from the MR Egger intercepts and Cochran *Q* tests for the IVs of MS and NMO on EBV antibodies were greater than .05, indicating no evidence of horizontal pleiotropy or heterogeneity. In addition, all *P*-values from the MR-PRESSO Global test were greater than .05, indicating the absence of outlying SNPs (Tables S15 and S16, Supplemental Digital Content, https://links.lww.com/MD/R626). The leave-one-out analysis confirmed the robustness of the findings, with the results remaining stable regardless of the exclusion of any single SNP (see Figs. S16–S25, Supplemental Digital Content, https://links.lww.com/MD/R628). To evaluate the plausibility of the reverse causal hypothesis, Steiger directionality tests were performed with the data used for the reverse GSMR analysis (MS→EBV antibodies). The results unequivocally rejected the reverse path, demonstrating that the genetic instruments explained significantly more variance in the EBV antibodies than in MS (all *P* < .01; see Table S17, Supplemental Digital Content, https://links.lww.com/MD/R627). This conclusion was further substantiated by a sensitivity analysis using conditionally selected instruments (GCTA-COJO and Steiger filtering), which was feasible only for the MS-to-EBV antibodies analysis (see Fig. S26, Supplemental Digital Content, https://links.lww.com/MD/R628). This indicates that the observed reverse MR associations are highly unlikely to represent a true causal relationship and are more likely attributable to shared genetic pleiotropy.

## 4. Discussion

Previous studies have revealed differential expression of EBV-related antibodies in MS, suggesting a potential role for these antibodies in the disease. Our study, using a genetic epidemiology approach, confirms and refines this relationship. While our primary GSMR analysis identified a significant association between elevated EBNA-1 antibody levels and increased MS risk, further investigation revealed critical nuance to this finding.

Applying this tiered interpretation framework, and considering the robustness to sensitivity analyses, the strength of association (*P* = 2.967 × 10^−6^), and its nominal replication in the IMSGC cohort, we classify the causal role of ZEBRA antibodies as high confidence. In contrast, the association for EBNA-1 is considered suggestive, as it was nominally supported in only one validation cohort (UKB) and was substantially attenuated after excluding HLA-region SNPs. Our initial observation that high anti-EBNA-1 antibody levels increase MS risk (OR = 1.284, *P* = 2.450 × 10^−4^) aligns with prior epidemiological findings. However, a key strength of the MR design is its ability to probe the robustness of such associations. When we performed a sensitivity analysis excluding the 2 strongest instrumental SNPs (rs6927022 and rs9273857) located in the HLA region, the association was substantially attenuated and became nonsignificant (OR = 0.927, *P* = .422). This indicates that the observed genetic correlation is largely mediated by shared genetic variation within the HLA region, suggesting pleiotropy (where the same genes influence both traits) rather than a direct causal effect of the antibody itself.

This finding provides a crucial genetic perspective for interpreting the extensive body of prior research linking EBV serology to MS. EBV infects and persists in human B cells by exploiting the B cell environment to sustain its life cycle while evading the host immune system through the minimal expression of viral proteins.^[28]^ Several EBV-specific antibodies can be detected in EBV carriers. These proteins include EBNA-1 (BKRF1), a latency protein; VCA p18 (BFRF3), a structural protein of the mature virus; ZEBRA (BZLF1), a lytic cycle protein; and EA-D, a basic component of the viral DNA replication complex.^[28,29]^ High antibody levels against EBNA-1 and VCA p18 (or VCA) are associated with an increased MS risk. In individuals with genetic factors associated with low EBNA-1 IgG titers, MS onset is delayed by 3.5 years.^[29–31]^

A region within the carboxyl half of EBNA-1 (amino acids 385–420) induces elevated antibody levels in patients with MS and shows the strongest association with MS risk among antibodies against other EBNA-1 domains.^[30]^ EBNA-1-specific antibodies have also been detected in the cerebrospinal fluid of some patients with MS. These antibodies may contribute to the formation of oligoclonal bands, which are produced by clonal B cell-derived plasma cells in the CNS and are a hallmark of MS.^[31]^ Injection of a peptide derived from the aa395–420 region of EBNA-1 into mice with experimental autoimmune encephalomyelitis exacerbated CNS autoimmunity.^[31,32]^

The asymmetric replication patterns observed for EBNA-1→MS and ZEBRA→MS associations across validation cohorts warrant careful consideration. Several methodological factors may contribute to these discrepancies. First, phenotype heterogeneity across datasets likely plays a significant role. The IMSGC cohort represents a deeply phenotyped multiple sclerosis specialist cohort, while the UKB cohort represents a broader population. This phenotypic spectrum difference may differentially affect associations with specific EBV antibody responses. Second, variations in instrument sets between primary and replication analyses emerged from our use of cohort-appropriate LD reference panels. The FinnGen primary analysis used European-specific LD references, while validation cohorts employed their respective genetic backgrounds, potentially introducing heterogeneity in IV selection. Third, exposure scaling differences across GWAS datasets, particularly in antibody measurement methodologies and normalization approaches, may have influenced effect size estimates and replication success.

Our genetic evidence suggests that these well-documented serological and experimental findings may be downstream manifestations of a shared genetic predisposition, primarily orchestrated through the HLA region. The prevailing pathogenic hypotheses, such as molecular mimicry and dysregulated immune surveillance, are fundamentally dependent on HLA-mediated antigen presentation and immune regulation. The molecular mimicry hypothesis posits that antibodies against VCA p18 cross-react with the cytoplasmic protein septin-9.^[33]^ Septin-9, which forms cytoplasmic filaments, participates in cytokinesis, exocytosis, neurite outgrowth, and cell cycle control. While expressed across various tissues, septin-9 is emerging as a potential autoantigen in MS owing to gene mutations associated with hereditary neuralgic amyotrophy.^[34]^ In the CNS, septin-9 primarily exists in neurons, and its expression is upregulated after trauma.^[35]^ Antibodies against EBNA-1 cross-react with anoctamin 2, ɑB-crystallin, myelin basic protein (MBP), and glial cell adhesion molecule.^[30,31,36]^ EBNA-1 shares structural homology with the CNS protein GlialCAM, particularly in the EBNA-1386-405 and GlialCAM370-389 regions.^[37]^ Anti-EBNA1 antibodies recognize GlialCAM epitopes and trigger complement-dependent oligodendrocyte cytotoxicity, with in vitro experiments demonstrating a >60% MBP degradation rate.^[38]^ EBNA1-specific CD4^+^ T cells, when presented via HLA-DRB1 * 15:01, cross-recognize myelin antigens (e.g. MOG35-55), driving Th17 polarization (3.2-fold increase in IL-17A secretion).^[38,39]^

Meanwhile, the dysregulated immune surveillance hypothesis stipulates that cytotoxic NKG2C+ and NKG2D+ natural killer (NK) cells eliminate GlialCAM-specific B and T cells. Patients with MS exhibit reduced NKG2C+ cell activity, leading to persisting autoreactive cells.^[37]^ In MS, autoreactive B cells upregulate HLA-E, which engages inhibitory NKG2A receptors on NK cells, shielding them against destruction. EBV latent membrane protein 1 variants (e.g. GGDPHLPTL) enhance HLA-E expression, exacerbating immune escape.^[37]^ Thus, the cross-reactive immune responses and defective viral control previously described are mechanistically rooted in the genetic functions of the HLA region. Our data demonstrate that this shared genetic architecture is the primary driver of the observed association, positioning EBNA-1 antibody levels as a downstream immunological phenotype of an underlying HLA-mediated dysregulation.

It is critical to define the empirical genetic associations discovered here from the mechanistic hypotheses they may support. Our suggestive-level evidence for the EBNA-1→MS association, which is heavily confounded by the HLA region, provides a genetic corollary for the long-standing mechanistic hypothesis of molecular mimicry. The observed genetic pleiotropy suggests that the well-documented serological cross-reactivity (e.g. between EBNA-1 and GlialCAM) may be a downstream consequence of a shared genetic predisposition that governs both MS risk and the anti-EBV immune response, rather than an independent causal pathway. Conversely, our high-confidence evidence for a protective effect of ZEBRA antibodies is a robust empirical finding. Although the mechanism is not yet defined, this finding supports the hypothesis that ZEBRA antibodies may neutralize or inhibit the viral protein, suppressing its deleterious effects in MS. This association warrants targeted experimental investigation to uncover the underlying biology.

Finally, our findings for non-MS ANDs (NMO, MG, GBS, and CIDP) are inconclusive due to limited statistical power (see Table S10, Supplemental Digital Content, https://links.lww.com/MD/R627). This means that we cannot currently support or refute any causal role of EBV antibodies in these conditions. We emphasize that these null results do not constitute evidence of absence; rather, they highlight the need for future studies with larger, well-powered cohorts to establish or refute empirical associations before mechanistic links can be meaningfully explored. The asymmetric replication patterns observed for EBNA-1→MS and ZEBRA→MS associations across validation cohorts warrant careful consideration. Several methodological factors may contribute to these discrepancies. First, phenotype heterogeneity across datasets likely plays a significant role. The IMSGC cohort represents a deeply phenotyped multiple sclerosis specialist cohort, while the UKB cohort represents a broader population. This phenotypic spectrum difference may differentially affect associations with specific EBV antibody responses. Second, variations in instrument sets between primary and replication analyses emerged from our use of cohort-appropriate LD reference panels. The FinnGen primary analysis used European-specific LD references, while validation cohorts employed their respective genetic backgrounds, potentially introducing heterogeneity in IV selection. Third, exposure scaling differences across GWAS datasets, particularly in antibody measurement methodologies and normalization approaches, may have influenced effect size estimates and replication success.

Notably, the protective association of ZEBRA antibodies with MS risk remained robust in our primary FinnGen analysis and was nominally validated in the IMSGC cohort (but not in UKB). This association persisted after rigorous sensitivity analyses. While the lack of replication in UKB suggests potential heterogeneity or cohort-specific factors, the overall evidence – including high statistical significance and robustness – supports a high-confidence, albeit not universally replicated, causal effect. This suggests that it may represent a more direct, non-HLA-mediated effect. ZEBRA is a viral immediate early gene that plays a crucial role in the transition of EBV from latency to lytic replication.^[40]^ Our study suggests that ZEBRA antibodies exert a protective effect in MS. BZLF1 (ZEBRA) has been detected in 46% of MS brain samples, primarily in association with chronic lesions, and in 44% of non-MS brain tissues.^[41]^ The reactivation of latent EBV infection has been linked to MS disease activity; however, this remains controversial.^[42–44^^]^ Previous research has suggested a deleterious role for ZEBRA in MS, but the role of antibodies against this antigen in MS remains poorly understood. The observed protective association of ZEBRA antibody levels with MS risk suggests a potential role for the lytic cycle antigen in disease pathogenesis. Further experimental investigation is warranted to elucidate the underlying biology.

The current study found no significant causal effects of EBV-specific antibodies on MG, GBS, or CIDP. However, these null findings should be interpreted with caution due to the limited statistical power (see Table S10, Supplemental Digital Content, https://links.lww.com/MD/R627). Further, we cannot rule out pathogenic roles for these antibodies. Confirmation in larger studies would suggest distinct pathogenic mechanisms across different ANDs and the need for targeted therapeutic strategies. This study employed a bidirectional MR design to dissect the complex relationship between EBV antibodies and MS. The discordance between GSMR and TSMR results in the reverse MR analyses indicate the need for a cautious biological interpretation.

The incorporation of Steiger directionality tests provided decisive evidence to clarify the causal direction. Importantly, under both the forward and reverse analytical frameworks, the Steiger test results were consistent and unequivocal, supporting a single causal direction from EBV antibodies to MS (all *P* < 1.0 × 10^−80^, see Table S14, Supplemental Digital Content, https://links.lww.com/MD/R627; all *P* < .01, see Table S17, Supplemental Digital Content, https://links.lww.com/MD/R627). This finding is significant on 2 fronts. First, it substantially strengthens the credibility of our forward MR results (antibodies→MS). Second, it clearly reveals that the observed reverse MR associations (MS→antibodies) are highly unlikely to represent a true biological causal pathway. Consequently, the most plausible explanation for the significant reverse GSMR results is shared genetic pleiotropy: common genetic variants, primarily in the HLA region, independently regulate both MS susceptibility and the intensity of the humoral immune response to EBV infection.

Methodological implications for reverse MR interpretation: The discordance between reverse GSMR (significant) and reverse TSMR (non-significant) underscores that such associations are method-dependent and may be sensitive to LD structure and pleiotropy handling. While GSMR identified signals likely reflecting shared genetic pathways, TSMR did not corroborate them as causal. This reinforces that reverse causality should not be concluded based on a single MR method, especially when directionality tests strongly favor the opposite causal path. Thus, our study illustrates the importance of integrating multiple analytical approaches and explicit directionality validation in MR to avoid misinterpretation of pleiotropic patterns as causal effects.

This study has several strengths. First, we effectively reduced the influence of confounding factors and improved the accuracy and reliability of our findings by using a novel MR method. Second, our comprehensive approach enabled an in-depth exploration of causal relationships between EBV antibodies and ANDs, providing a detailed understanding of their interactions. Third, the use of the GSMR approach helped reduce the impact of pleiotropy and minimized potential confounding effects. Finally, as all GWAS data were sourced from Europeans, the risk of population stratification bias in the results is minimal. Additionally, a comprehensive set of sensitivity analyses, including conditional IV selection and Steiger filtering for directionality, were implemented to address potential methodological concerns. These rigorous checks confirmed that our principal findings concerning the causal roles of EBNA-1 and ZEBRA antibodies in MS are remarkably robust and not susceptible to biases from weak instruments, pleiotropy, or reverse causation.

Several limitations warrant consideration. First, the use of summary-level data constrained our ability to detect non-linear effects. Second, MR analyses for non-MS autoimmune neurological diseases (NMO, MG, GBS, CIDP) were statistically underpowered (Table S17, Supplemental Digital Content, https://links.lww.com/MD/R627), precluding definitive conclusions. Third, the exclusive European ancestry of our data limits generalizability to other populations. Fourth, our analyses were based on a single LD reference panel (1000 Genomes European). While the use of alternative LD panels (e.g., UKB-based LD) might influence instrument selection, our comprehensive sensitivity analyses demonstrate that the core causal inferences – particularly the protective effect of ZEBRA antibodies on MS – are robust and unlikely to be driven by LD-specific idiosyncrasies. Future studies could further validate our findings using multiple LD reference sets to enhance generalizability. Although we employed methods (e.g., MR-PRESSO) to mitigate pleiotropy, residual confounding from complex genetic pathways, especially within the MHC/HLA region, cannot be fully ruled out. Future studies with individual-level data, diverse cohorts, and non-HLA instruments are needed for validation.

In conclusion, our study provides strong genetic evidence for a directional association between EBV-specific antibody levels and MS risk, with ZEBRA antibodies showing a robust protective effect independent of HLA confounding. Supported by strong evidence from Steiger directionality tests, we establish that the primary causal direction is from EBV antibodies to MS, though we acknowledge that residual pleiotropy and LD-related uncertainties warrant cautious interpretation. Within this pathway, the risk effect of EBNA-1 antibodies is largely driven by a shared genetic background mediated by the HLA region. Meanwhile, ZEBRA antibodies demonstrate a protective effect independent of HLA. The associations observed in the reverse analyses reflect the aforementioned genetic pleiotropy, not reverse causation. These findings underscore the potential causal role of EBV in MS etiology and highlight the importance of the genetic basis governing the anti-EBV immune response, while encouraging further validation in independent cohorts and with alternative LD reference panels. For other ANDs (NMO, MG, GBS, CIDP), our study lacked statistical power to draw definitive conclusions. Future studies with larger sample sizes are needed to clarify whether EBV antibodies play a causal role in these conditions.

## Acknowledgments

The authors thank the Multiple Sclerosis Genetics Consortium, International Myasthenia Gravis Genomics Consortium, UK Biobank, and FinnGen for providing publicly available GWAS summary statistics for MS, MG, and GBS for this analysis. We also thank GWAS summary statistics for antibody immune responses to 5 types of herpesvirus and NMO, MG, and CIDP.

## Author contributions

**Conceptualization:** Anding Xu.

**Formal analysis:** Shujun Sun, Yiyong Wen.

**Funding acquisition:** Shujun Sun, Anding Xu.

**Methodology:** Shujun Sun.

**Project administration:** Anding Xu.

**Supervision:** Anding Xu.

**Validation:** Shujun Sun.

**Visualization:** Shujun Sun.

**Writing – original draft:** Shujun Sun, Yiyong Wen.

**Writing – review & editing:** Yiyong Wen, Anding Xu, Tao Ding.

## Supplementary Material







## References

[1] Khan AW Farooq M Hwang MJ Haseeb M Choi S. Autoimmune neuroinflammatory diseases: role of interleukins. Int J Mol Sci. 2023;24:7960. 10.3390/ijms24097960 PMC10178921 37175665

[2] Bhagavati S. Autoimmune disorders of the nervous system: pathophysiology, clinical features, and therapy. Front Neurol. 2021;12:664664. 10.3389/fneur.2021.664664 PMC8079742 33935958

[3] Zarobkiewicz MK Morawska I Michalski A Roliński J Bojarska-Junak A. NKT and NKT-like cells in autoimmune neuroinflammatory diseases-multiple sclerosis, myasthenia gravis and Guillain–Barré syndrome. Int J Mol Sci. 2021;22:9520. 10.3390/ijms22179520 PMC8431671 34502425

[4] Gogia B Rocha Cabrero F Khan Suheb MZ Lui F Rai PK. Chronic Inflammatory Demyelinating Polyradiculoneuropathy. StatPearls Publishing Copyright © 2024, StatPearls Publishing LLC.; 2024. 33085396

[5] Miller FW. The increasing prevalence of autoimmunity and autoimmune diseases: an urgent call to action for improved understanding, diagnosis, treatment, and prevention. Curr Opin Immunol. 2023;80:102266. 10.1016/j.coi.2022.102266 PMC9918670 36446151

[6] Pisetsky DS. Pathogenesis of autoimmune disease. Nat Rev Nephrol. 2023;19:509–24. 10.1038/s41581-023-00720-1 PMC10171171 37165096

[7] Bjornevik K Münz C Cohen JI Ascherio A. Epstein–Barr virus as a leading cause of multiple sclerosis: mechanisms and implications. Nat Rev Neurol. 2023;19:160–71. 10.1038/s41582-023-00775-5 36759741

[8] Bjornevik K Cortese M Healy BC. Longitudinal analysis reveals high prevalence of Epstein–Barr virus associated with multiple sclerosis. Science. 2022;375:296–301. 10.1126/science.abj8222 35025605

[9] DeLorenze GN Munger KL Lennette ET Orentreich N Vogelman JH Ascherio A. Epstein–Barr virus and multiple sclerosis: evidence of association from a prospective study with long-term follow-up. Arch Neurol. 2006;63:839–44. 10.1001/archneur.63.6.noc50328 16606758

[10] Shi Z Kong L Wang R. Cytomegalovirus and Epstein–Barr virus infections in patients with neuromyelitis optica spectrum disorder. J Neurol. 2024;271:6089–95. 10.1007/s00415-024-12571-2 39046523

[11] Wahren B Link H. Antibodies to Epstein–Barr virus and cytomegalovirus in Guillain–Barré syndrome. J Neurol Sci. 1976;28:129–38. 10.1016/0022-510x(76)90098-8 178829

[12] Lünemann JD Tackenberg B Stein A. Dysregulated Epstein–Barr virus infection in patients with CIDP. J Neuroimmunol. 2010;218:107–11. 10.1016/j.jneuroim.2009.11.003 19939466

[13] Kakalacheva K Maurer MA Tackenberg B Münz C Willcox N Lünemann JD. Intrathymic Epstein–Barr virus infection is not a prominent feature of myasthenia gravis. Ann Neurol. 2011;70:508–14. 10.1002/ana.22488 21905082

[14] Cavalcante P Marcuzzo S Franzi S. Epstein–Barr virus in tumor-infiltrating b cells of myasthenia gravis thymoma: an innocent bystander or an autoimmunity mediator? Oncotarget. 2017;8:95432–49. 10.18632/oncotarget.20731 PMC5707033 29221139

[15] Smith GD Ebrahim S. ‘Mendelian randomization’: can genetic epidemiology contribute to understanding environmental determinants of disease? Int J Epidemiol. 2003;32:1–22. 10.1093/ije/dyg070 12689998

[16] Zheng J Baird D Borges MC. Recent developments in Mendelian randomization studies. Curr Epidemiol Rep. 2017;4:330–45. 10.1007/s40471-017-0128-6 PMC5711966 29226067

[17] Lin BD Alkema A Peters T. Assessing causal links between metabolic traits, inflammation and schizophrenia: a univariable and multivariable, bidirectional Mendelian-randomization study. Int J Epidemiol. 2019;48:1505–14. 10.1093/ije/dyz176 PMC7070229 31504541

[18] Butler-Laporte G Kreuzer D Nakanishi T Harroud A Forgetta V Richards JB. Genetic determinants of antibody-mediated immune responses to infectious diseases agents: a genome-wide and HLA association study. Open Forum Infect Dis. 2020;7:ofaa450. 10.1093/ofid/ofaa450 PMC7641500 33204752

[19] Patsopoulos NA Baranzini SE Santaniello A. Multiple sclerosis genomic map implicates peripheral immune cells and microglia in susceptibility. Science. 2019;365:eaav7188. 10.1126/science.aav7188 PMC7241648 31604244

[20] Estrada K Whelan CW Zhao F. A whole-genome sequence study identifies genetic risk factors for neuromyelitis optica. Nat Commun. 2018;9:1929. 10.1038/s41467-018-04332-3 PMC5955905 29769526

[21] Jiang L Zheng Z Fang H Yang J. A generalized linear mixed model association tool for Biobank-scale data. Nat Genet. 2021;53:1616–21. 10.1038/s41588-021-00954-4 34737426

[22] Wang K Zhang Q Zhang P Yang Q Pan F Zha B. Use of bidirectional mendelian randomization to unveil the association of helicobacter pylori infection and autoimmune thyroid diseases. Sci Adv. 2024;10:eadi8646. 10.1126/sciadv.adi8646 PMC11290481 39083605

[23] Shim H Aspichueta P Chasman DI. A multivariate genome-wide association analysis of 10 LDL subfractions, and their response to statin treatment, in 1868 Caucasians. PLOS ONE 2015;10:e0120758. 10.1371/journal.pone.0120758 PMC4405269 25898129

[24] Zhu Z Zheng Z Zhang F. Causal associations between risk factors and common diseases inferred from GWAS summary data. Nat Commun. 2018;9:224. 10.1038/s41467-017-02317-2 PMC5768719 29335400

[25] Sekula P Del Greco M F Pattaro C. Mendelian randomization as an approach to assess causality using observational data. J Am Soc Nephrol. 2016;27:3253–65. 10.1681/ASN.2016010098 PMC5084898 27486138

[26] Sedgwick P. Multiple hypothesis testing and Bonferroni’s correction. BMJ. 2014;349:g6284. 10.1136/bmj.g6284 25331533

[27] Burgess S. Sample size and power calculations in mendelian randomization with a single instrumental variable and a binary outcome. Int J Epidemiol. 2014;43:922–9. 10.1093/ije/dyu005 PMC4052137 24608958

[28] Middeldorp JM Brink AA van den Brule AJ Meijer CJ. Pathogenic roles for Epstein–Barr virus (EBV) gene products in EBV-associated proliferative disorders. Crit Rev Oncol Hematol. 2003;45:1–36. 10.1016/s1040-8428(02)00078-1 12482570

[29] Zhang Q Holley-Guthrie E Ge JQ Dorsky D Kenney S. The Epstein–Barr virus (EBV) DNA polymerase accessory protein, BMRF1, activates the essential downstream component of the EBV oriLyt. Virology. 1997;230:22–34. 10.1006/viro.1997.8470 9126259

[30] Bray PF Luka J Bray PF Culp KW Schlight JP. Antibodies against Epstein–Barr nuclear antigen (EBNA) in multiple sclerosis CSF, and two pentapeptide sequence identities between EBNA and myelin basic protein. Neurology. 1992;42:1798–804. 10.1212/wnl.42.9.1798 1381067

[31] Lanz TV Brewer RC Ho PP. Clonally expanded B cells in multiple sclerosis bind EBV EBNA1 and GlialCAM. Nature. 2022;603:321–7. 10.1038/s41586-022-04432-7 PMC9382663 35073561

[32] Kyllesbech C Trier N Slibinskas R. Virus-specific antibody indices may supplement the total IgG index in diagnostics of multiple sclerosis. J Neuroimmunol. 2022;367:577868. 10.1016/j.jneuroim.2022.577868 35477126

[33] Lindsey JW. Antibodies to the Epstein–Barr virus proteins BFRF3 and BRRF2 cross-react with human proteins. J Neuroimmunol. 2017;310:131–4. 10.1016/j.jneuroim.2017.07.013 28778437

[34] Kuhlenbäumer G Hannibal MC Nelis E. Mutations in SEPT9 cause hereditary neuralgic amyotrophy. Nat Genet. 2005;37:1044–6. 10.1038/ng1649 16186812

[35] Mao H Liu J Shi W. The expression patterns of septin-9 after traumatic brain injury in rat brain. J Mol Neurosci. 2013;51:558–66. 10.1007/s12031-013-0024-6 23700216

[36] Tengvall K Huang J Hellström C. Molecular mimicry between anoctamin 2 and Epstein–Barr virus nuclear antigen 1 associates with multiple sclerosis risk. Proc Natl Acad Sci U S A. 2019;116:16955–60. 10.1073/pnas.1902623116 PMC6708327 31375628

[37] Vietzen H Berger SM Kühner LM. Ineffective control of Epstein–Barr-virus-induced autoimmunity increases the risk for multiple sclerosis. Cell. 2023;186:5705–18.e13. 10.1016/j.cell.2023.11.015 38091993

[38] Sattarnezhad N Kockum I Thomas OG. Antibody reactivity against EBNA1 and GlialCAM differentiates multiple sclerosis patients from healthy controls. Proc Natl Acad Sci U S A. 2025;122:e2424986122. 10.1073/pnas.2424986122 PMC11929495 40063790

[39] Saggau C Bacher P Esser D. Autoantigen-specific CD4(+) T cells acquire an exhausted phenotype and persist in human antigen-specific autoimmune diseases. Immunity. 2024;57:2416–32.e8. 10.1016/j.immuni.2024.08.005 39226901

[40] Osipova-Goldberg HI Turchanowa LV Adler B Pfeilschifter JM. H2O2 INHIBITS BCR-dependent immediate early induction of EBV genes in Burkitt’s lymphoma cells. Free Radic Biol Med. 2009;47:1120–9. 10.1016/j.freeradbiomed.2009.06.019 19540913

[41] Moreno MA Or-Geva N Aftab BT. Molecular signature of Epstein–Barr virus infection in MS brain lesions. Neurol Neuroimmunol Neuroinflamm. 2018;5:e466. 10.1212/NXI.0000000000000466 PMC5994704 29892607

[42] Maple PAC Gran B Tanasescu R Pritchard DI Constantinescu CS. An absence of Epstein–Barr virus reactivation and associations with disease activity in people with multiple sclerosis undergoing therapeutic hookworm vaccination. Vaccines (Basel). 2020;8:487. 10.3390/vaccines8030487 PMC7564729 32872342

[43] Torkildsen O Nyland H Myrmel H Myhr KM. Epstein–Barr virus reactivation and multiple sclerosis. Eur J Neurol. 2008;15:106–8. 10.1111/j.1468-1331.2007.02009.x 18042233

[44] Yea C Tellier R Chong P. Epstein-Barr virus in oral shedding of children with multiple sclerosis. Neurology. 2013;81:1392–9. 10.1212/WNL.0b013e3182a841e4 PMC3806908 24014504

