# Supplementary material for: Causal effects of Epstein–Barr virus antibodies on autoimmune neuroinflammatory diseases: A generalised summary data-based Mendelian randomisation study
Source: Medicine (Baltimore). 2026 Apr 10;105(15):e48264. doi: 10.1097/MD.0000000000048264 (PMC13593266; doi:10.1097/MD.0000000000048264)
Supplement: Supplementary file 1 [file medi-105-e48264-s002.pdf]

Supplemental Digital Content Table 7. Cohort-Leave-Out Sensitivity Analysis: Robustness of Causal Effects of EBV Antibodies on MS.

**Exposure**	**Outcome**	**GSMR (main analysis)**		**MR Egger**		**Weighted median**		**Inverse variance weighted**	
		**OR (95%CI)**	***P***	**OR(95%CI)**	**P**	**OR (95%CI)**	***P***	**OR (95%CI)**	***P***
EBV EBNA-1 antibody levels	MS (FinnGen)	0.927 (0.770-1.116)	0.422	1.112(0.723-1.711)	0.638	1.120(0.855-1.466)	0.410	1.111(0.908- 1.360)	0.305
EBV ZEBRA antibody levels		/[a]	/[a]	/[b]	/[b]	/[b]	/[b]	/[b]	/[b]

Notes: This table presents the causal estimates of EBV EBNA-1 and ZEBRA antibody levels on MS (FinnGen) from the GSMR after sequentially excluding instrumental variables identified in the FinnGen cohorts. a: The "/" symbol indicates results identical to those in Table 2, as the SNP significantly associated with MS (e.g., rs67838634) had already been excluded during the HEIDI-outlier filtering in the GSMR analysis. b：The "/" symbol indicates results identical to those in Table 2, as rs67838634 had already been excluded during during pleiotropy screening in the MR-PRESSO tests.

Abbreviations: CI, confidence interval; EBNA, Epstein–Barr virus nuclear antigen 1; EBV, Epstein–Barr Virus; GSMR, generalized summary-data-based Mendelian randomization; MS, multiple sclerosis; OR, odds ratio; ZEBRA, Z EBV replication activator.

Supplemental Digital Content Table 8. Causal effects of Epstein-Barr virus antibodies on autoimmune neuroinflammatory diseases: Robustness analysis with enhanced instrumental variable screening

Exposure	Outcome	OR (95% CI)	P-value	SNPs	Mean_F	Steiger_Success_Rate
EBV EA-D antibody levels	MS	0.787 (0.610-1.015)	0.065	7/6/4	42.840	1.000
	NMO	0.691 (0.184-2.599)	0.585	7/3/3	35.027	1.000
	MG	0.764 (0.493-1.182)	0.226	7/6/6	42.840	1.000
	GBS	1.235 (0.790-1.929)	0.355	7/6/6	42.840	1.000
	CIDP	1.012 (0.677-1.514)	0.953	7/7/7	43.791	1.000
EBV EBNA-1 antibody levels	MS	1.807 (1.574-2.074)	4.176e-17	35/26/12	64.244	0.824
	NMO	1.199 (0.737-1.951)	0.465	35/14/14	52.473	1.000
	MG	1.006 (0.840-1.204)	0.950	35/26/26	64.244	1.000
	GBS	0.927 (0.771-1.115)	0.422	35/26/26	64.244	1.000
	CIDP	0.935 (0.804-1.088)	0.386	35/31/31	69.493	1.000
EBV VCA p18 antibody levels	CIDP	1.324 (0.802-2.186)	0.272	4/3/3	70.209	1.000
	MS	0.598 (0.501-0.712)	9.676e-09	15/9/5	68.755	1.000

Exposure	Outcome	OR (95% CI)	P-value	SNPs	Mean_F	Steiger_Success_Rate
EBV ZEBRA antibody levels	NMO	0.240 (0.075-0.766)	0.016	15/5/5	32.547	0.833
	MG	0.659 (0.474-0.914)	0.013	15/9/9	68.755	1.000
	GBS	0.694 (0.496-0.971)	0.033	15/9/9	68.755	1.000
	CIDP	0.895 (0.710-1.127)	0.346	15/12/12	81.837	1.000

Abbreviations: MS: Multiple sclerosis; NMO: Neuromyelitis optica; MG: Myasthenia gravis; GBS: Guillain-Barré syndrome; CIDP: Chronic inflammatory demyelinating polyneuropathy; EBV: Epstein-Barr virus; EA-D: Early antigen-D; EBNA-1: EBV nuclear antigen-1; VCA p18: Viral capsid antigen p18; ZEBRA: Z EBV replication activator; OR: Odds ratio; CI: Confidence interval; SNP: Single nucleotide polymorphism.

Supplemental Digital Content Table 9. Instrumental variable robustness assessment for Mendelian randomisation analyses.

Exposure	Outcome	COJO_Independent_SNPs	After_Harmonisation	After_Steiger	After_F_filter	After_HEIDI	Mean_F_before_pruning	Median_F_before_pruning	Mean_F_after_pruning	Median_F_after_pruning	Min_F_after_pruning	All_F_above_10	Steiger_Success_Rate	HEIDI_used	MR_PRESSO_Global_P
EBV EA-D antibody levels	MS	7	6	6	6	4	42.84	38.217	42.84	38.217	30.963	TRUE	1	TRUE	NA
EBV EA-D antibody levels	NMO	7	3	3	3	3	35.027	32.471	35.027	32.471	30.963	TRUE	1	TRUE	NA
EBV EA-D antibody levels	MG	7	6	6	6	6	42.84	38.217	42.84	38.217	30.963	TRUE	1	TRUE	0.551
EBV EA-D antibody levels	GBS	7	6	6	6	6	42.84	38.217	42.84	38.217	30.963	TRUE	1	TRUE	0.375
EBV EA-D antibody levels	CIDP	7	7	7	7	7	43.791	41.649	43.791	41.649	30.963	TRUE	1	TRUE	0.255
EBV EBNA-1 antibody levels	MS	39	28	28	28	10	61.122	43.739	61.122	43.739	21.211	TRUE	1	TRUE	NA
EBV EBNA-1 antibody levels	NMO	39	18	15	15	15	51.582	40.821	51.528	35.76	21.211	TRUE	0.833	TRUE	0.341
EBV EBNA-1 antibody levels	MG	39	28	28	28	28	61.122	43.739	61.122	43.739	21.211	TRUE	1	TRUE	0.1
EBV EBNA-1 antibody levels	GBS	39	28	28	28	28	61.122	43.739	61.122	43.739	21.211	TRUE	1	TRUE	NA
EBV EBNA-1 antibody levels	CIDP	39	32	32	32	32	67.852	46.668	67.852	46.668	21.211	TRUE	1	TRUE	0.644
EBV VCA p18 antibody levels	MS	4	4	4	4	4	61.378	61.371	61.378	61.371	34.884	TRUE	1	FALSE	NA
EBV VCA p18 antibody levels	MG	4	4	4	4	4	61.378	61.371	61.378	61.371	34.884	TRUE	1	FALSE	0.8
EBV VCA p18 antibody levels	GBS	4	4	4	4	4	61.378	61.371	61.378	61.371	34.884	TRUE	1	FALSE	0.488
EBV VCA p18 antibody levels	CIDP	4	3	3	3	3	70.209	69.445	70.209	69.445	53.296	TRUE	1	TRUE	NA
EBV ZEBRA	MS	15	9	9	9	4	69.178	39.043	69.178	39.043	21.501	TRUE	1	TRUE	NA

antibody levels															
EBV ZEBRA antibody levels	NMO	15	6	4	4	4	35.355	35.237	31.874	28.455	21.501	TRUE	0.667	FALSE	0.19
EBV ZEBRA antibody levels	MG	15	9	9	9	9	69.178	39.043	69.178	39.043	21.501	TRUE	1	TRUE	0.982
EBV ZEBRA antibody levels	GBS	15	9	9	9	9	69.178	39.043	69.178	39.043	21.501	TRUE	1	TRUE	0.228
EBV ZEBRA antibody levels	CIDP	15	11	11	11	11	86.074	45.592	86.074	45.592	21.501	TRUE	1	TRUE	0.993

Abbreviations: COJO: Conditional and joint analysis; SNP: Single nucleotide polymorphism; F filter: F-statistic filter (F > 10 indicates strong instruments); HEIDI: Heterogeneity in dependent instruments; MR-PRESSO: Mendelian Randomization Pleiotropy RESidual Sum and Outlier; MS: Multiple sclerosis; NMO: Neuromyelitis optica; MG: Myasthenia gravis; GBS: Guillain-Barré syndrome; CIDP: Chronic inflammatory demyelinating polyneuropathy; EBV: Epstein-Barr virus; EA-D: Early antigen-D; EBNA-1: EBV nuclear antigen-1; VCA p18: Viral capsid antigen p18; ZEBRA: Z EBV replication activator.

Notes: This table summarizes the robustness assessment of instrumental variables used in the bidirectional Mendelian randomization analysis. All analyses used genetic instruments with F-statistics > 10, indicating strong instruments. The Steiger filtering success rate indicates the proportion of SNPs that correctly identified the exposure-outcome directionality. HEIDI outlier detection was applied to remove pleiotropic instruments. MR-PRESSO global test P-values > 0.05 indicate no significant horizontal pleiotropy.

Supplemental Digital Content Table 10. Power analysis for MR of non-multiple sclerosis AND.

EBV Antibody	Disease	Cases	Effective Sample Size†	Total R^2	Detectable OR Range‡	Power Status§	Interpretation
**Anti-EBV IgG seropositivity**	NMO	215	733	0.0082	0.04 - 24.95	**Severely Underpowered**	Can only detect extremely large effects (OR > 24.9)
	MG	504	2,014	0.0107	1.40e-8 - 7.16e7	**Severely Underpowered**	Essentially no detection capability
	GBS	489	1,954	0.0107	8.67e-9 - 1.15e8	**Severely Underpowered**	Essentially no detection capability
	CIDP	425	1,698	0.0134	4.34e-9 - 2.30e8	**Severely Underpowered**	Essentially no detection capability
**EBV EA-D antibody levels**	NMO	215	733	0.0177	0.11 - 8.97	**Severely Underpowered**	Can only detect very large effects (OR > 8.97)
	MG	504	2,014	0.0381	7.00e-5 - 14,287	**Severely Underpowered**	Can only detect extreme effects
	GBS	489	1,954	0.0381	5.44e-5 - 18,390	**Severely Underpowered**	Can only detect extreme effects
	CIDP	425	1,698	0.0451	2.76e-5 -	**Severely**	Can only detect extreme

EBV Antibody	Disease	Cases	Effective Sample Size†	Total R^2	Detectable OR Range‡	Power Status§	Interpretation
					36,198	**Underpowered**	effects
**EBV EBNA-1 antibody levels**	NMO	215	733	0.0454	0.25 - 3.94	**Underpowered**	Can detect moderate to large effects (OR > 3.94)
	MG	504	2,014	0.1104	0.0036 - 275.27	**Underpowered**	Can detect large effects
	GBS	489	1,954	0.1104	0.0031 - 319.24	**Underpowered**	Can detect large effects
	CIDP	425	1,698	0.1164	0.0015 - 685.89	**Underpowered**	Can detect large effects
**EBV VCA p18 antibody levels**	NMO	215	733	0.0446	0.25 - 3.98	**Underpowered**	Can detect moderate to large effects (OR > 3.98)
	MG	504	2,014	0.0596	4.77e-4 - 2,095.77	**Severely Underpowered**	Can only detect extreme effects
	GBS	489	1,954	0.0596	3.90e-4 - 2,564.29	**Severely Underpowered**	Can only detect extreme effects
	CIDP	425	1,698	0.0638	1.48e-4 -	**Severely**	Can only detect extreme

EBV Antibody	Disease	Cases	Effective Sample Size†	Total R^2	Detectable OR Range‡	Power Status§	Interpretation
					6,756.51	**Underpowered**	effects
**EBV ZEBRA antibody levels**	NMO	215	733	0.0242	0.15 - 6.54	**Severely Underpowered**	Can only detect large effects (OR > 6.54)
	MG	504	2,014	0.0729	9.95e-4 - 1,005.45	**Severely Underpowered**	Can only detect extreme effects
	GBS	489	1,954	0.0729	8.29e-4 - 1,206.61	**Severely Underpowered**	Can only detect extreme effects
	CIDP	425	1,698	0.0700	2.20e-4 - 4,552.76	**Severely Underpowered**	Can only detect extreme effects

Notes:
†Effective sample size = 4 / (1/n_cases + 1/n_controls), accounting for case-control imbalance; ‡Detectable odds ratio range at 80% power and α = 0.05 (two-sided), representing the minimum effect sizes that could be detected; §Power status classification: Adequate (detectable OR < 1.5), Underpowered (1.5 ≤ detectable OR < 5), Severely Underpowered (detectable OR ≥ 5). Power calculations follow Burgess et al{Burgess, 2014 #360}. Severely underpowered analyses (detectable OR ⩾ 5) cannot reliably detect or rule out biologically plausible effects, and null findings should be interpreted as inconclusive.
Abbreviations: AND, Autoimmune Neuroinflammatory Diseases; MR, Mendelian Randomization; NMO, neuromyelitis optica; MG, myasthenia gravis; GBS, Guillain-Barré syndrome; CIDP, chronic inflammatory demyelinating polyneuropathy; EBV, Epstein-Barr virus; EBNA-1, EBV nuclear antigen 1; VCA, viral capsid antigen; ZEBRA, Z EBV replication activator; OR, odds ratio; R^2, proportion of variance explained by genetic instruments.

Supplemental Digital Content Table 13. MR-RAPS analysis for the causal effects of EBV EBNA-1 and ZEBRA antibody levels on MS risk.

Exposure	Outcome	Method	No. of SNPs	OR (95% CI)	P-value	Normality Test (Anderson-Darling)	Normality Test (Shapiro-Wilk)
EBV EBNA-1 antibody levels	MS	MR-RAPS	13	1.48 (1.29-1.70)	5.38e-08	A = 0.226, p = 0.770	W = 0.949, p = 0.585
EBV ZEBRA antibody levels	MS	MR-RAPS	11	0.64 (0.55-0.75)	1.81e-08	A = 0.419, p = 0.261	W = 0.931, p = 0.460

Note: ORs represent the change in MS risk per 1 standard deviation (SD) increase in genetically predicted antibody levels (standardized log-MFI). The non-significant p-values from the Anderson-Darling and Shapiro-Wilk normality tests (all > 0.05) indicate that the standardized residuals of the MR-RAPS models approximately follow a normal distribution, supporting the validity of the model assumptions.. CI, Confidence Interval; MR-RAPS, Mendelian Randomization using Robust Adjusted Profile Score; MS, Multiple Sclerosis; OR, Odds Ratio; SNP, Single Nucleotide Polymorphism.

Supplemental Digital Content Table 14. Steiger directionality tests for forward MR analysis of EBV antibodies on MS.

Exposure	Outcome	SNP R^2 (Exposure)	SNP R^2 (Outcome)	Correct Causal Direction	P-value
EBV EBNA-1 antibody levels	MS	0.11167	5.34×10^{-4}	True	7.89×10^{-174}
EBV VCA p18 antibody levels	MS	0.05972	1.41×10^{-3}	True	1.32×10^{-80}
EBV ZEBRA antibody levels	MS	0.07380	5.22×10^{-4}	True	1.49×10^{-112}

Footnote: Steiger directionality tests were performed on the harmonized dataset used for the forward GSMR analysis. The tests evaluate whether the genetic instruments explain more variance in the exposure than in the outcome. A result of "True" for "Correct Causal Direction" indicates that the instruments explain significantly more variance in the exposure than the outcome, providing strong statistical support for the causal direction from EBV antibodies to MS. All tests were statistically significant, robustly confirming the validity of the forward causal direction. SNP R^2 values represent the proportion of phenotypic variance explained by the genetic instruments. EBV, Epstein-Barr virus; EBNA-1, EBV nuclear antigen 1; VCA, viral capsid antigen; ZEBRA, Z EBV replication activator; MS, Multiple Sclerosis.

Supplemental Digital Content Table 17. Steiger directionality tests for reverse MR analysis of the effects of MS on EBV antibodies.

Exposure	Outcome	SNP R^2 (Exposure)	SNP R^2 (Outcome)	Correct Causal Direction	P-value
MS	EBV EBNA-1 antibody levels	0.00396	0.02245	False	2.18×10^{-14}
MS	EBV VCA p18 antibody levels	0.00396	0.01436	False	2.61×10^{-7}
MS	EBV ZEBRA antibody levels	0.00396	0.00866	False	7.63×10^{-3}

Note: Steiger directionality tests were performed on the harmonized dataset used for the reverse GSMR analysis. The tests evaluate whether the genetic instruments explain more variance in the exposure than in the outcome. A result of "False" for "Correct Causal Direction" indicates that the instruments explain significantly more variance in the outcome than the exposure, providing strong statistical evidence against the reverse causal direction and in favor of the opposite causal pathway. All tests were statistically significant, robustly rejecting the validity of the reverse causal direction. SNP R^2 values represent the proportion of phenotypic variance explained by the genetic instruments. EBNA-1, Epstein-Barr virus nuclear antigen 1; VCA, viral capsid antigen; ZEBRA, Z EBV replication activator; MS, Multiple Sclerosis.
