# Supplementary material for: Causal effects of Epstein–Barr virus antibodies on autoimmune neuroinflammatory diseases: A generalised summary data-based Mendelian randomisation study
Source: Medicine (Baltimore). 2026 Apr 10;105(15):e48264. doi: 10.1097/MD.0000000000048264 (PMC13593266; doi:10.1097/MD.0000000000048264)

Supplemental Digital Content Figure 1. Causal effects of Epstein-Barr virus antibodies on autoimmune neuroinflammatory diseases: GSMR analysis with CoJo-based instrument selection and Steiger directionality validation.

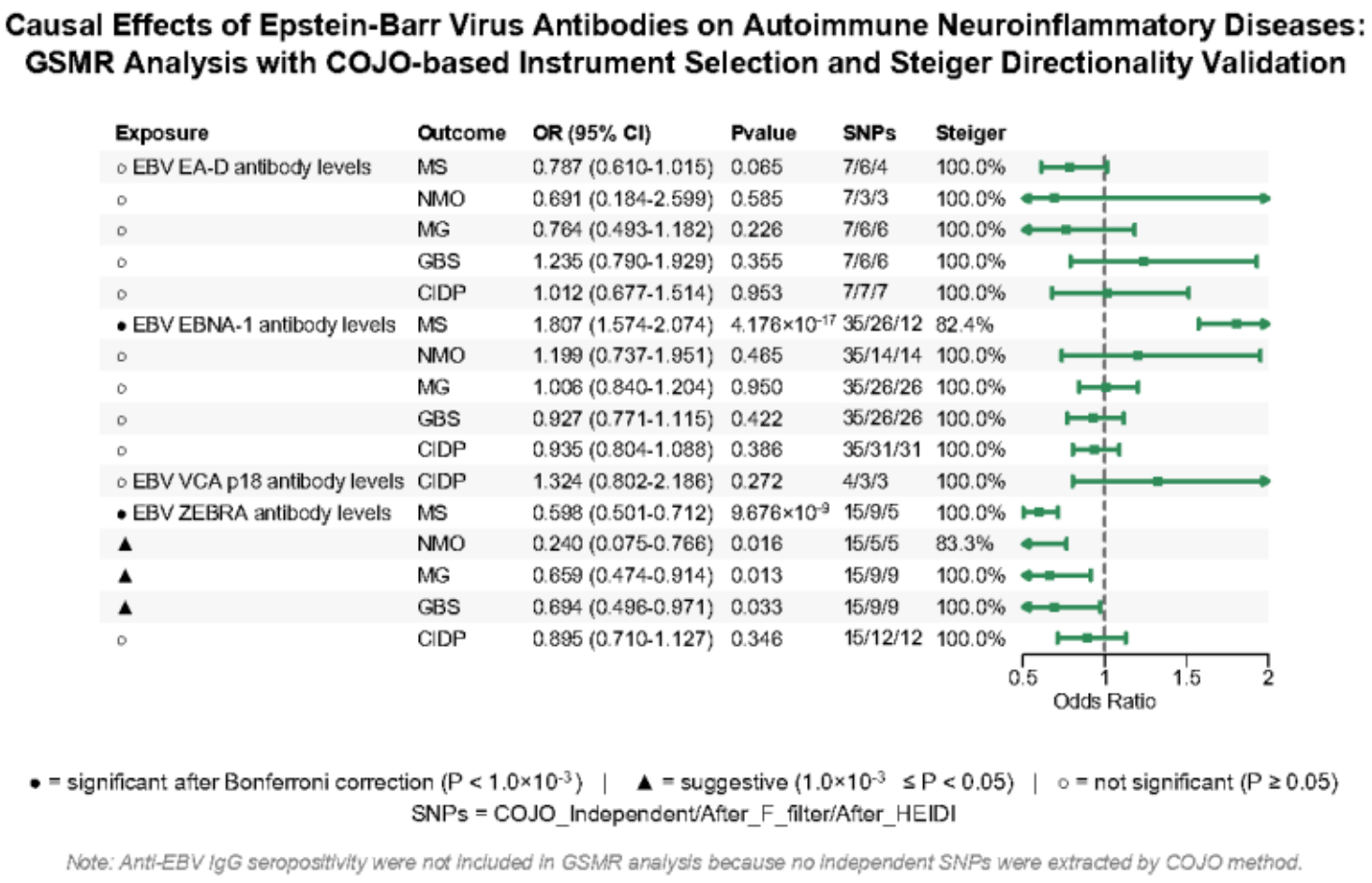

Supplemental Digital Content Figure 2. Forest plot displaying the TSMR analysis results for the effect of antibodies against EBV on ANDs. The analysis is conducted using TSMR, with a Bonferroni-corrected significance threshold set at $P = 1.0 \times 10^{-3}$

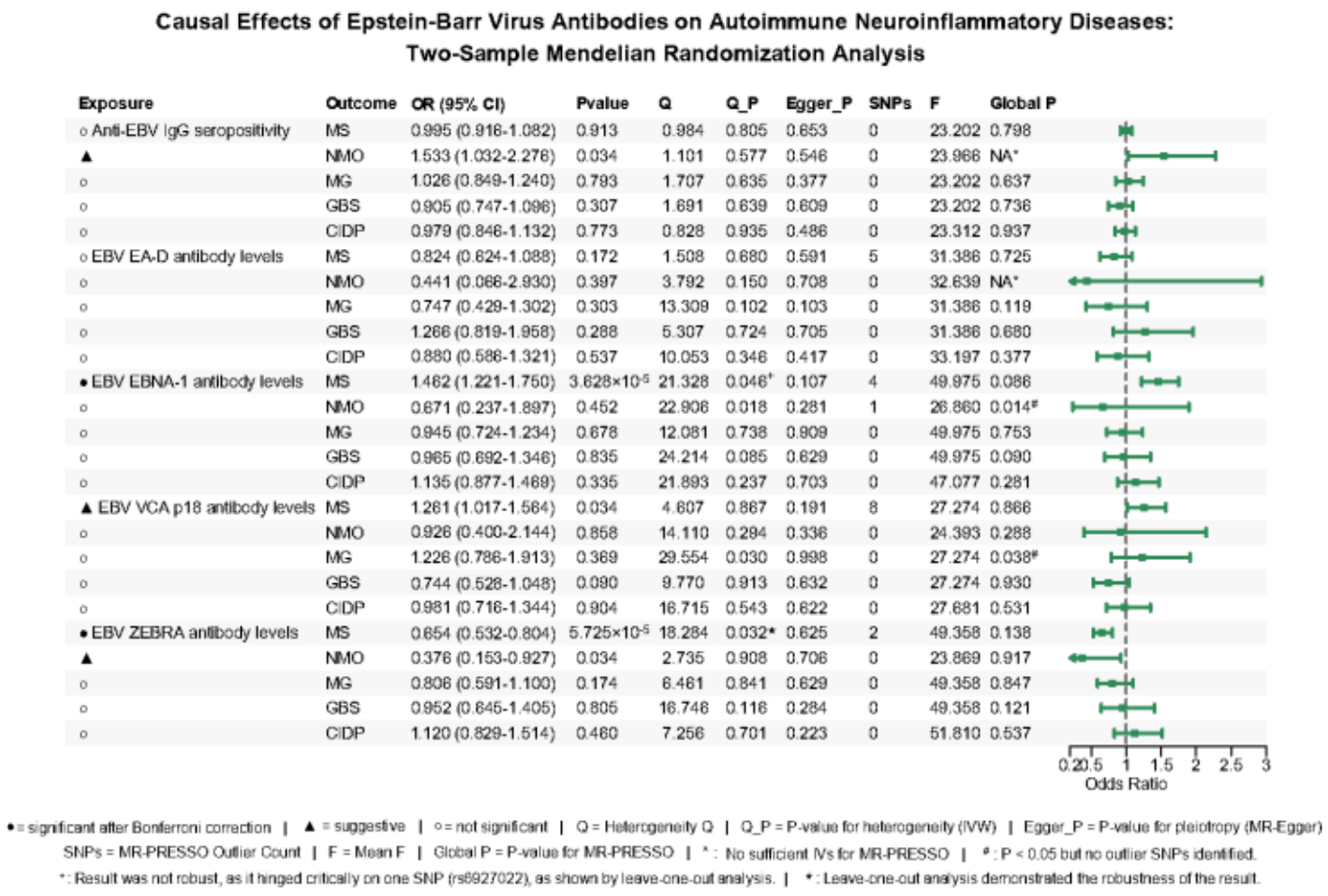

Supplemental Digital Content Figure 3. MR estimate results of the effect of anti-EBV IgG seropositivity on MS.

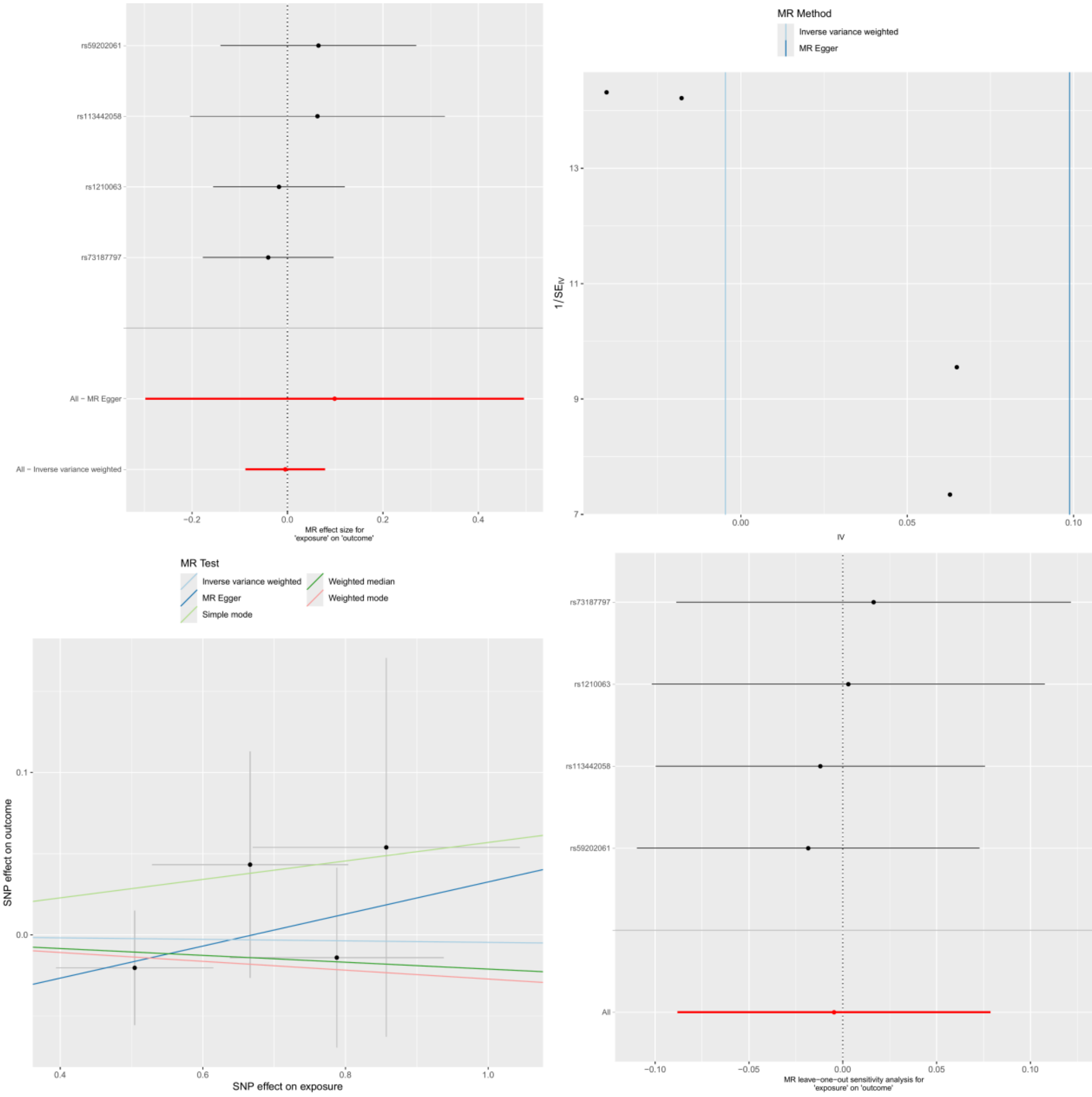

Supplemental Digital Content Figure 4. MR estimate results of the effect of EBV EA-D antibody levels on MS.

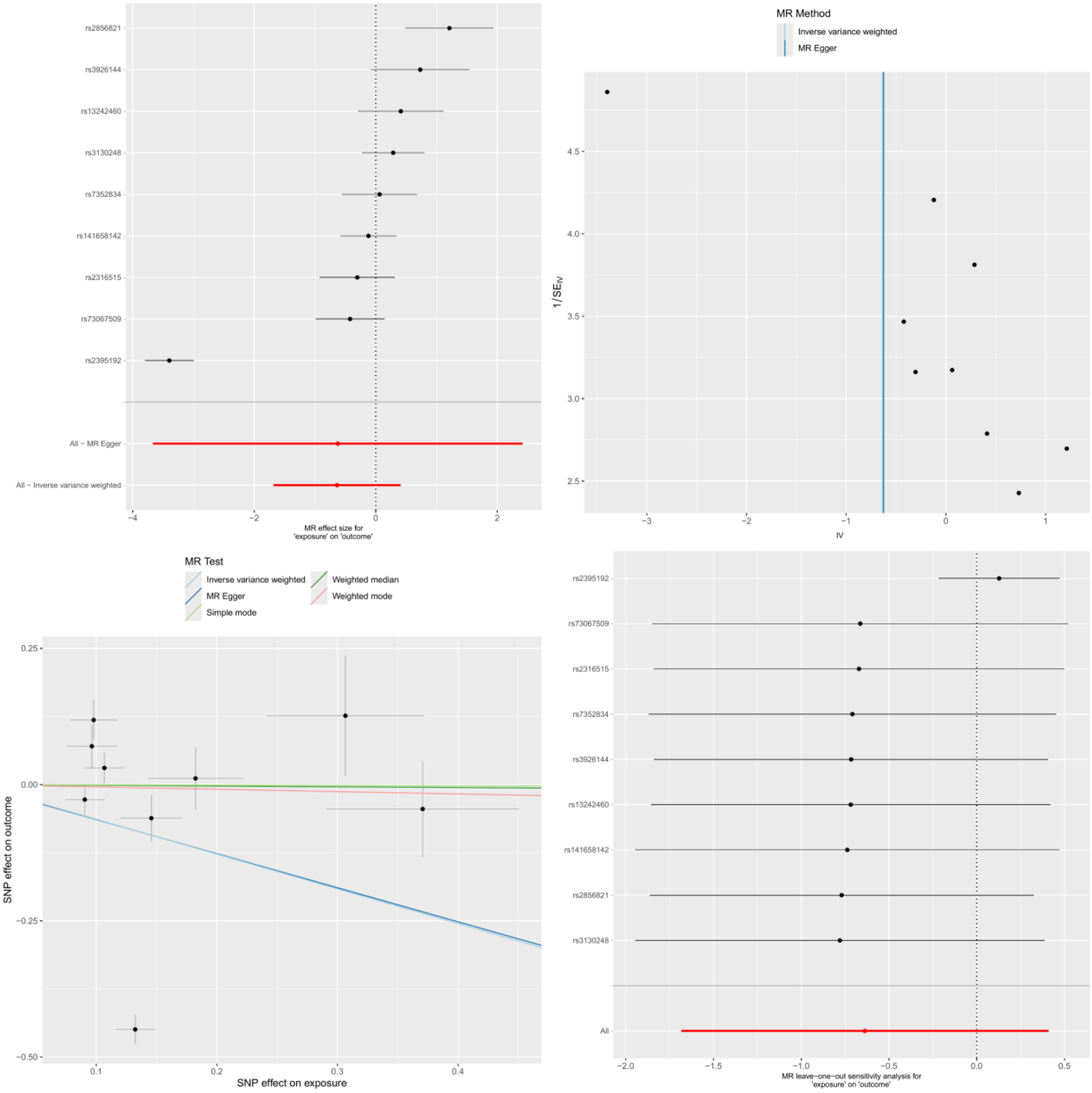

Supplemental Digital Content Figure 5. MR estimate results of the effect of EBV EBNA-1 antibody levels on MS.

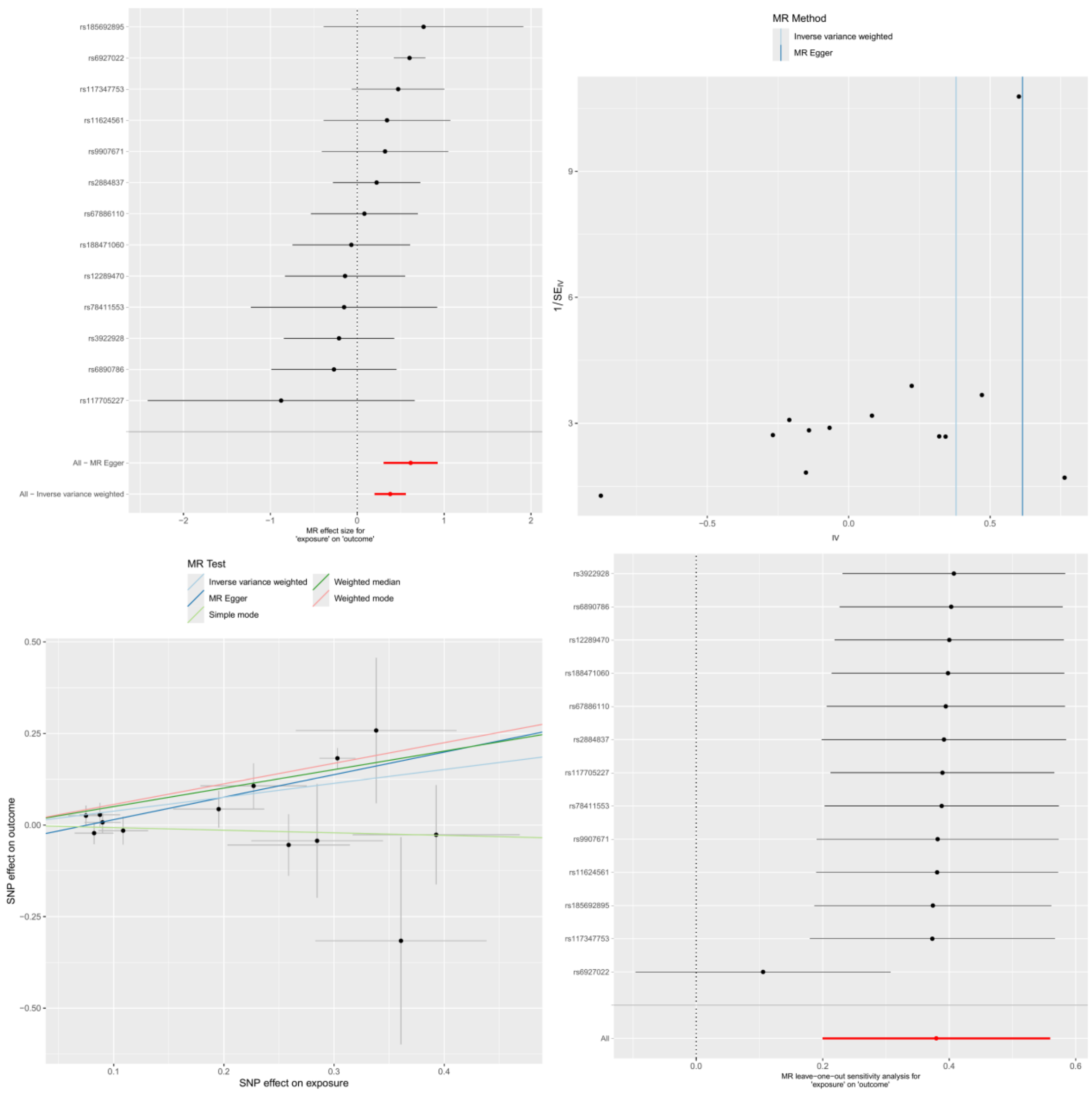

Supplemental Digital Content Figure 6. MR estimate results of the effect of EBV VCA p18 antibody levels on MS.

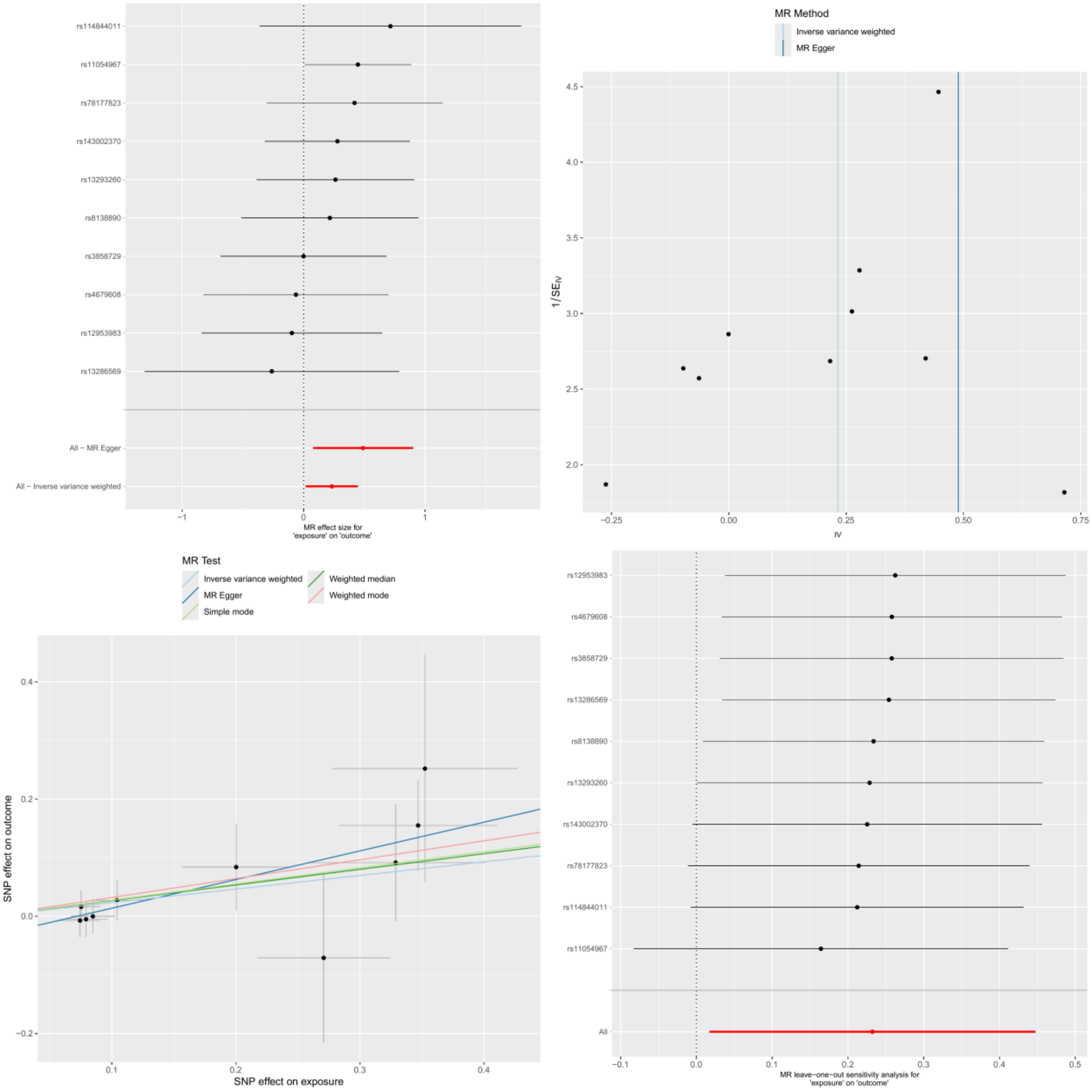

Supplemental Digital Content Figure 7. MR estimate results of the effect of EBV ZEBRA antibody levels on MS.

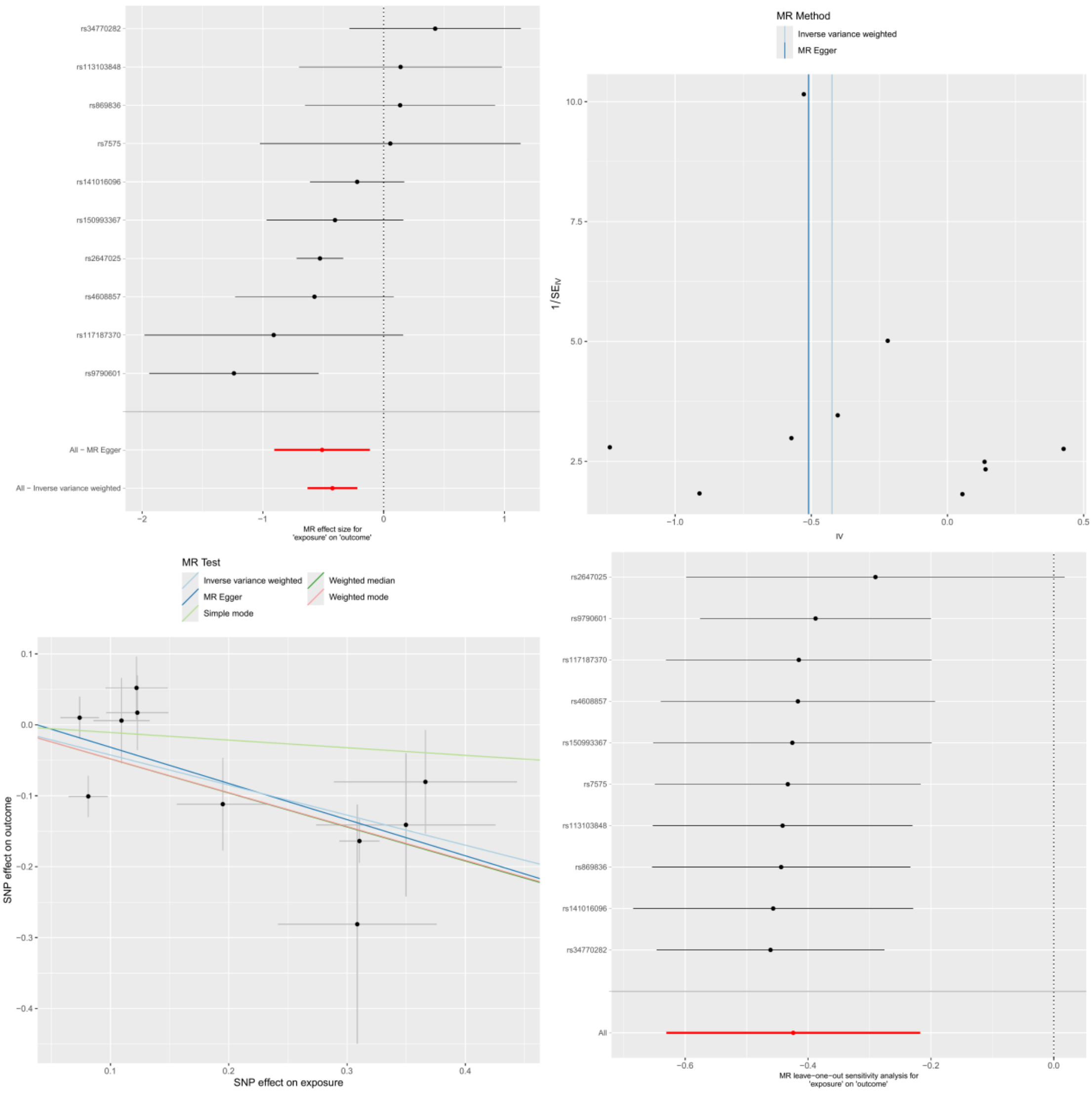

Supplemental Digital Content Figure 8. MR estimate results of the effect of anti-EBV IgG seropositivity on NMO.

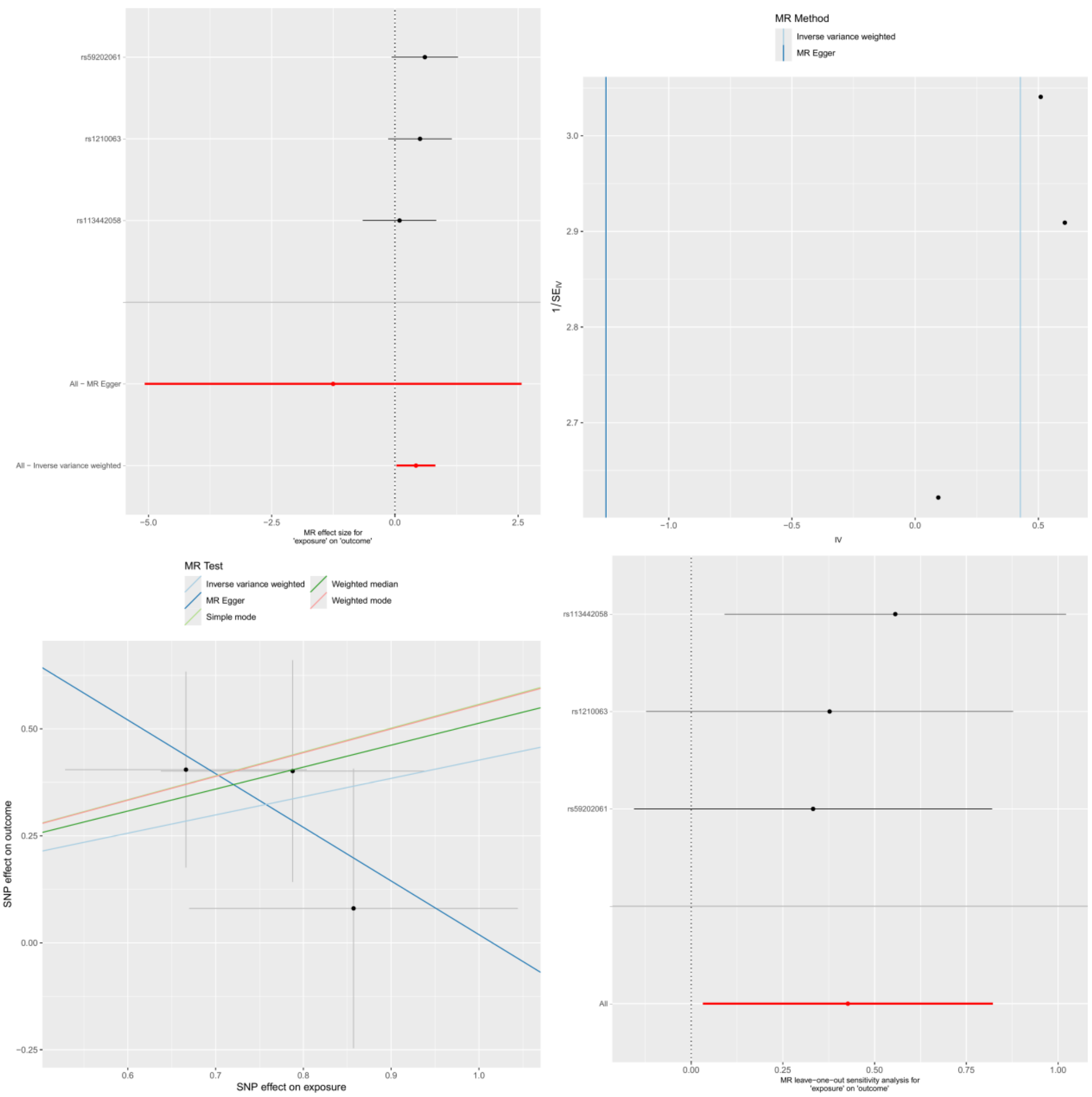

Supplemental Digital Content Figure 9. MR estimate results of the effect of EBV EA-D antibody levels on NMO.

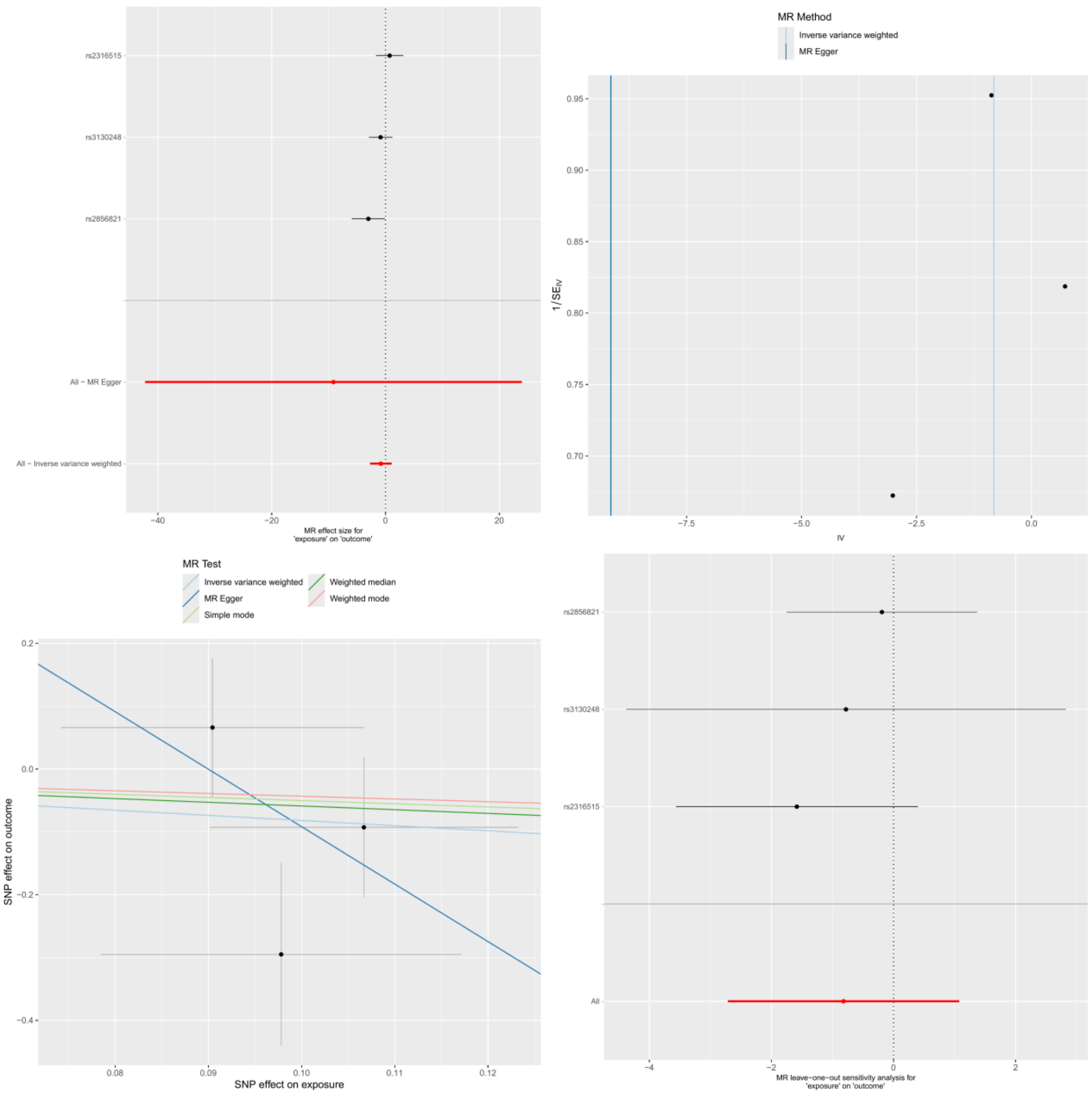

Supplemental Digital Content Figure 10. MR estimate results of the effect of EBV EBNA-1 antibody levels on NMO.

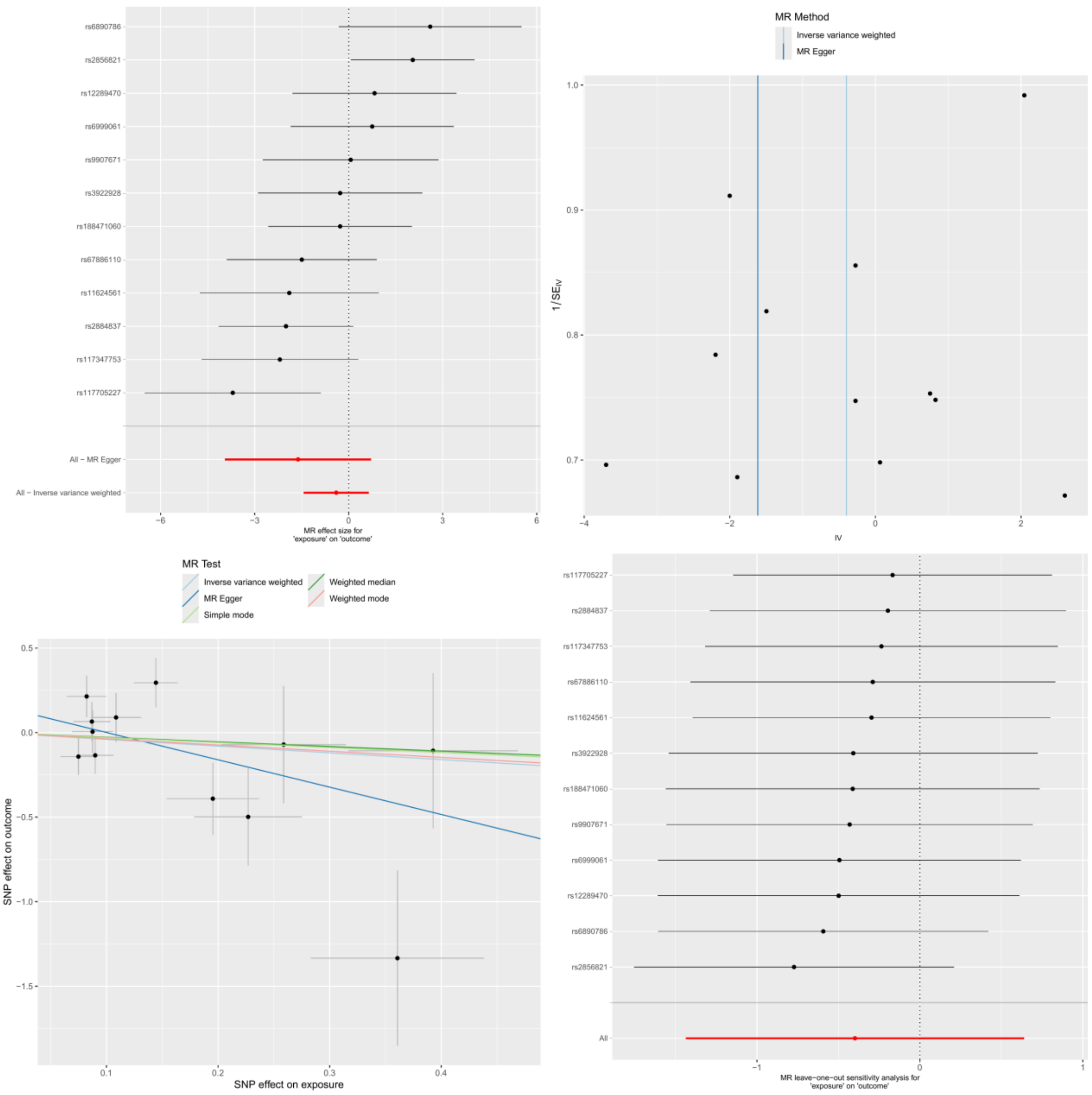

Supplemental Digital Content Figure 11. MR estimate results of the effect of EBV VCA p18 antibody levels on NMO.

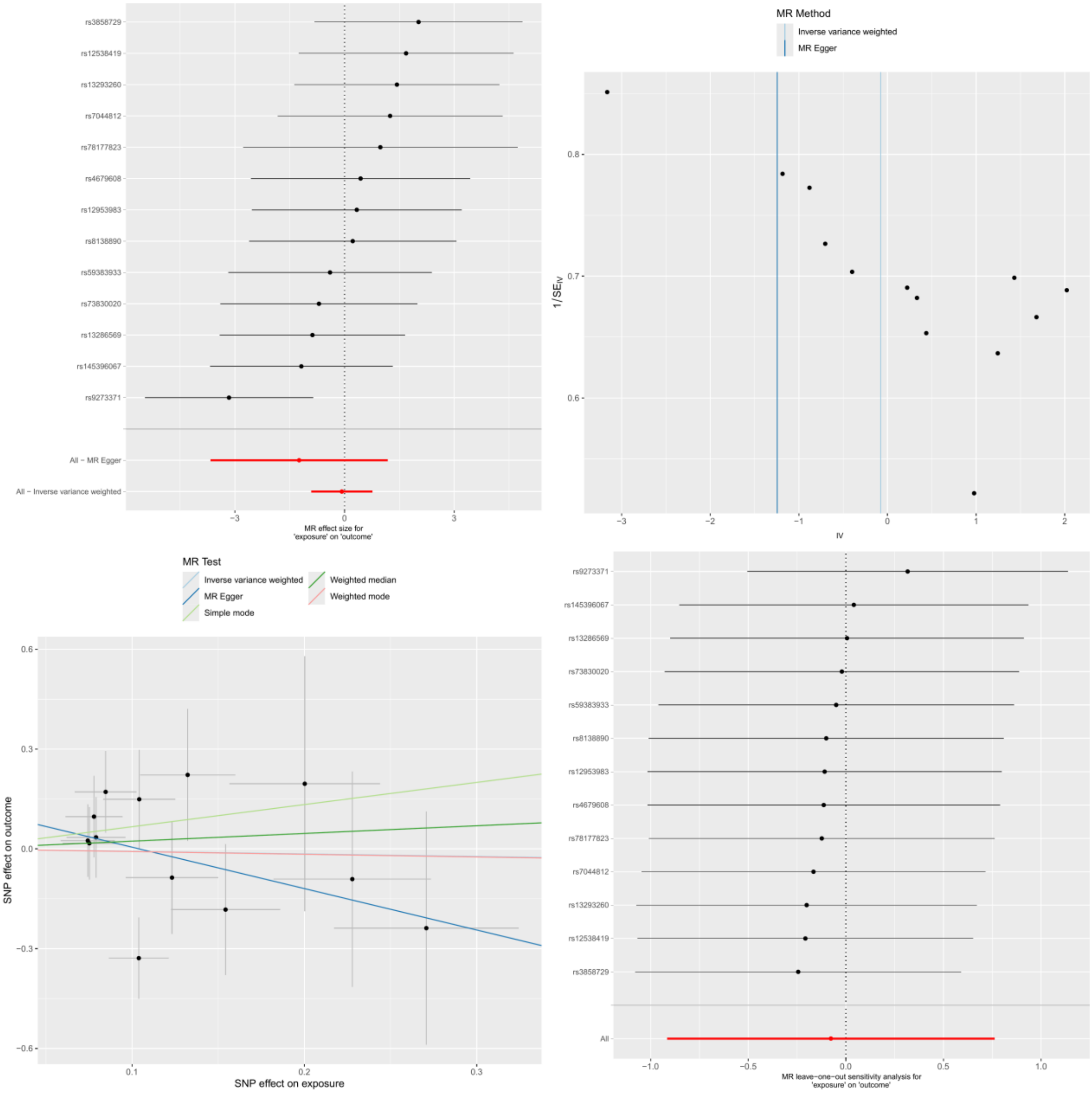

Supplemental Digital Content Figure 12. MR estimate results of the effect of EBV ZEBRA antibody levels on NMO.

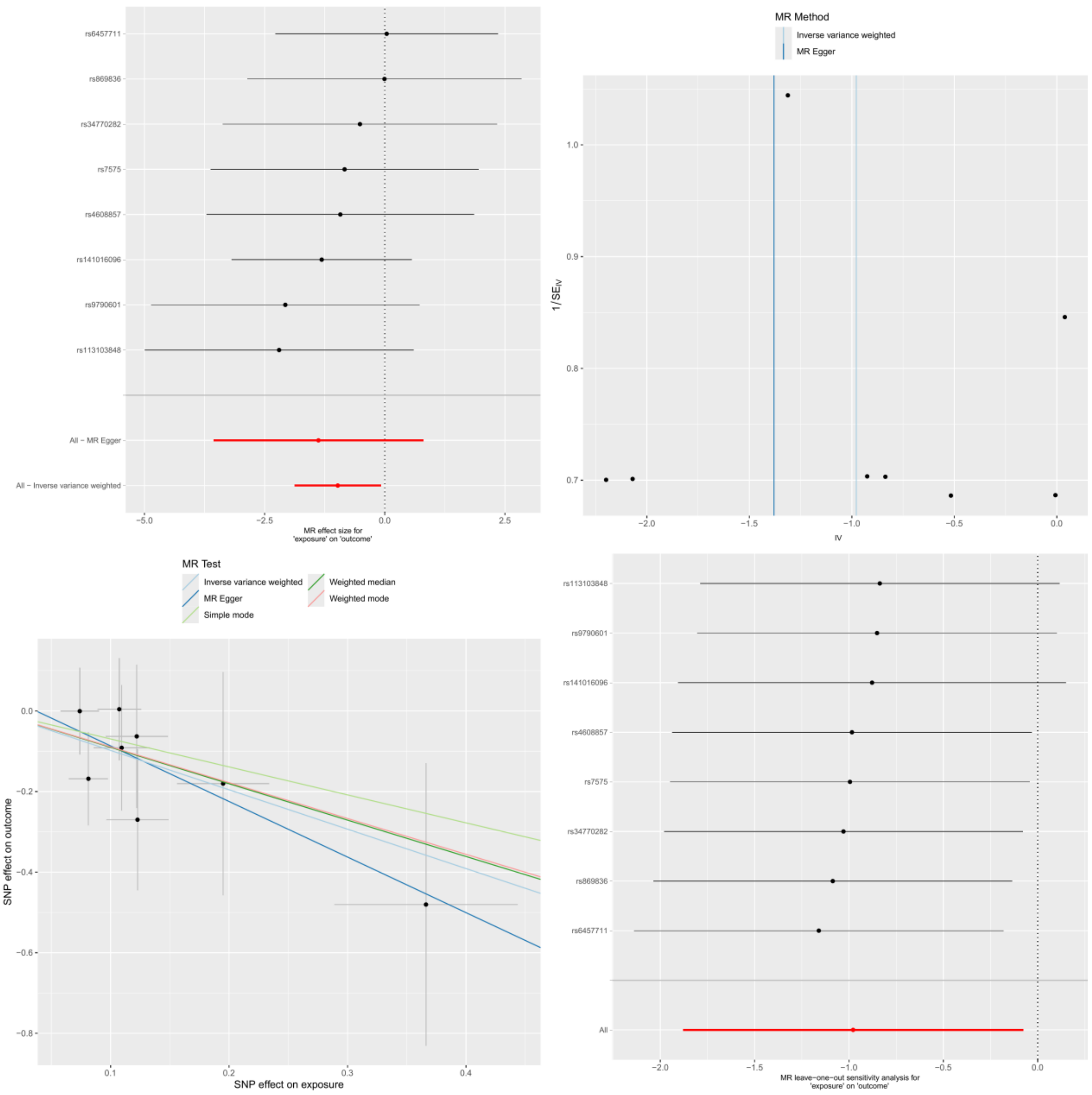

Supplemental Digital Content Figure 13. MR-RAPS analysis of the effects of EBNA-1 antibody levels on the risk of multiple sclerosis. (A) Leave-one-out sensitivity analysis: Effect estimates after excluding each SNP (blue dots). Most estimates cluster around the overall estimate (red line), but exclusion of rs6927022 shows notable deviation, suggesting this SNP has substantial influence on the results. (B) Funnel plot: Relationship between SNP effect sizes and precision shows approximately symmetric distribution, suggesting no substantial bias. Statistical analysis: MR-RAPS analysis reveal a significant positive association between EBNA-1 antibody levels and multiple sclerosis risk (OR = 1.479, 95% CI: 1.285-1.704, $P = 5.38\times10^{-8}$). Standardised residuals follow normal distribution ($P > 0.05$), supporting model validity.

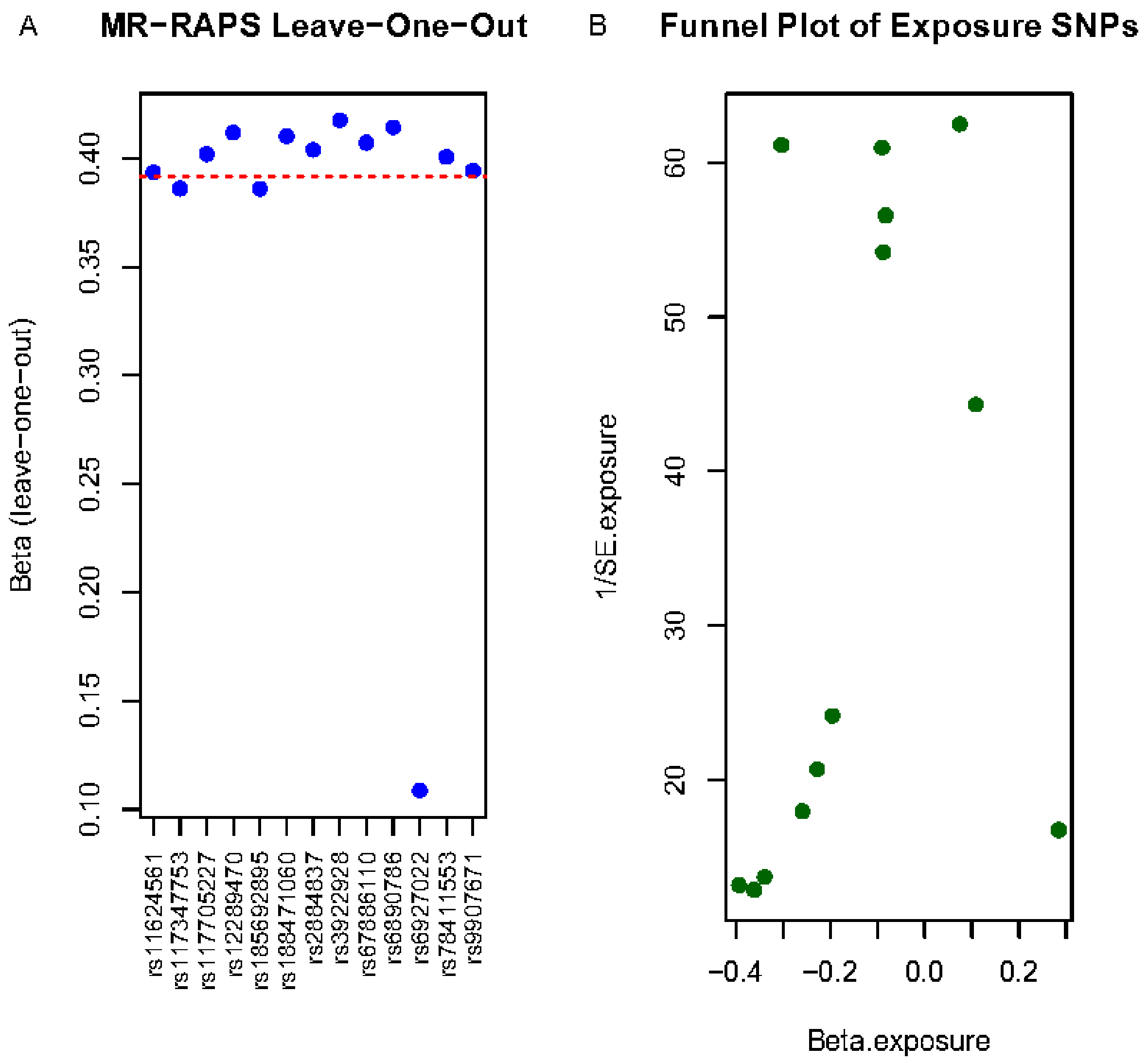

Supplemental Digital Content Figure 14. MR-RAPS analysis of the effects of ZEBRA antibody levels on multiple sclerosis risk. (A) Leave-one-out sensitivity analysis: Effect estimates after excluding each SNP (blue dots) cluster closely around the overall estimate (red line), indicating robust results. (B) Funnel plot: Relationship between SNP effect sizes and precision shows approximately symmetric distribution, suggesting no substantial bias. Statistical analysis: MR-RAPS analysis reveal a significant inverse association between ZEBRA antibody levels and multiple sclerosis risk (OR = 0.642, 95% CI: 0.551-0.749, P = 1.81×10^{-8}). Standardised residuals follow normal distribution (P > 0.05), supporting model validity.

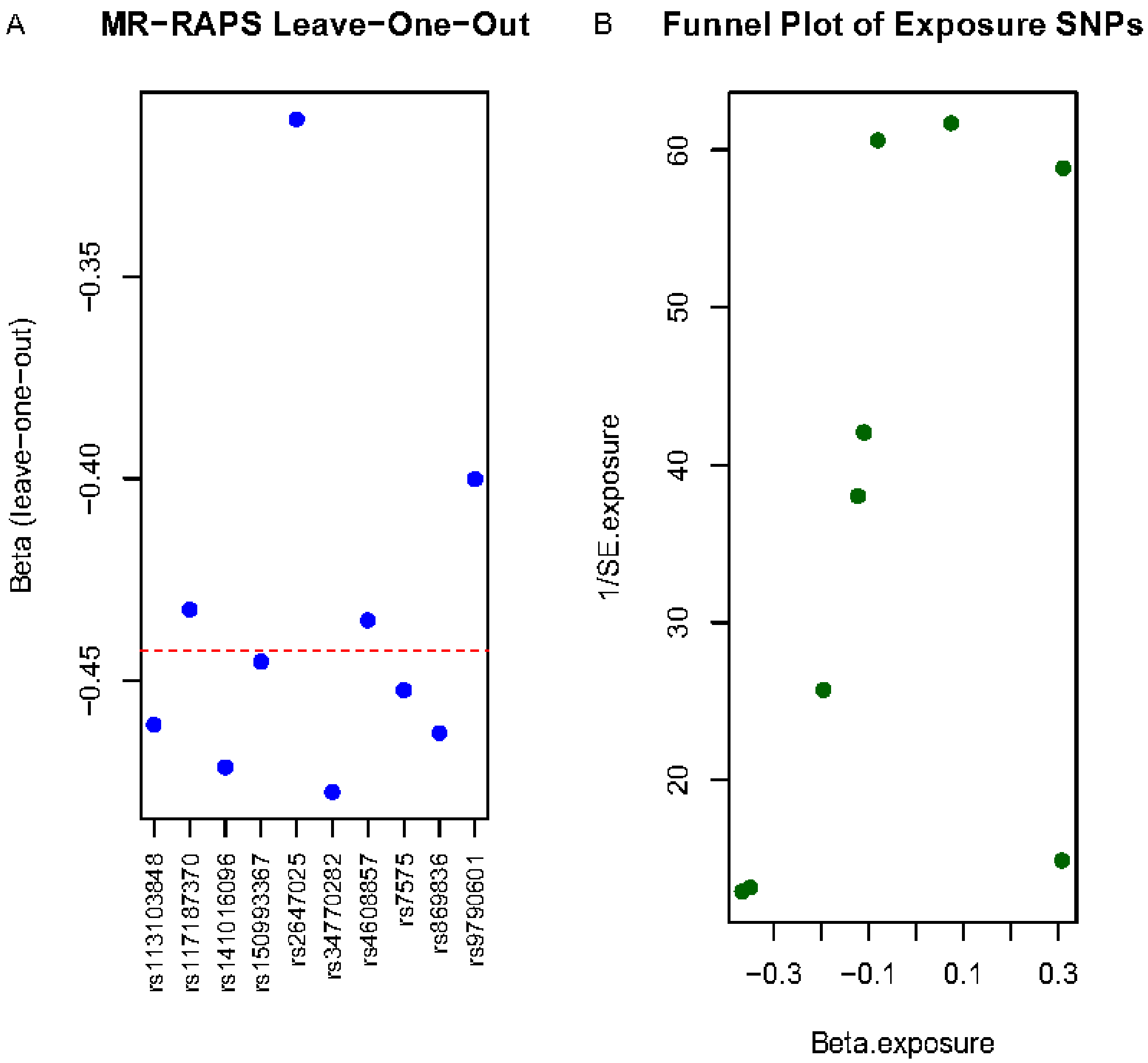

Supplemental Digital Content Figure 15. Forest plot displaying the TSMR analysis results for the effect of ANDs on EBV antibodies. The analysis is conducted using TSMR, with a Bonferroni-corrected significance threshold set at $P = 1.0 \times 10^{-3}$

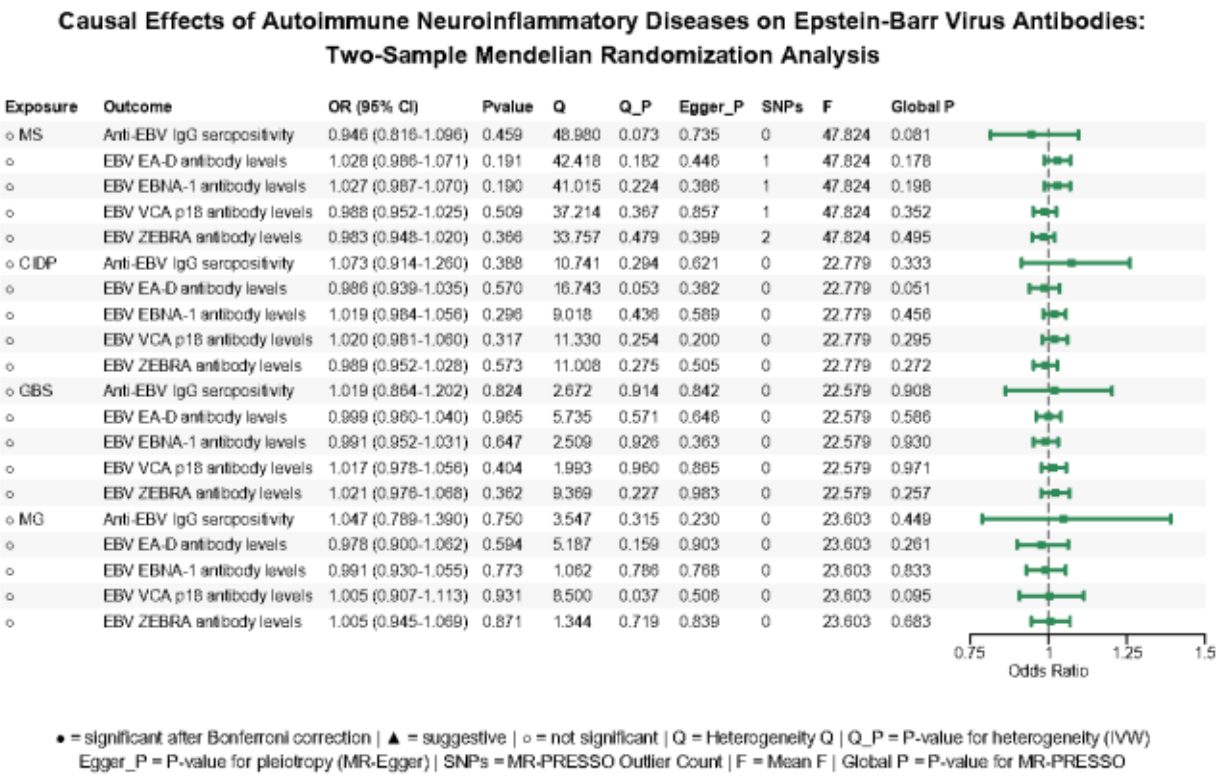

Supplemental Digital Content Figure 16. MR estimate results of the effects of MS on Anti-EBV IgG seropositivity.

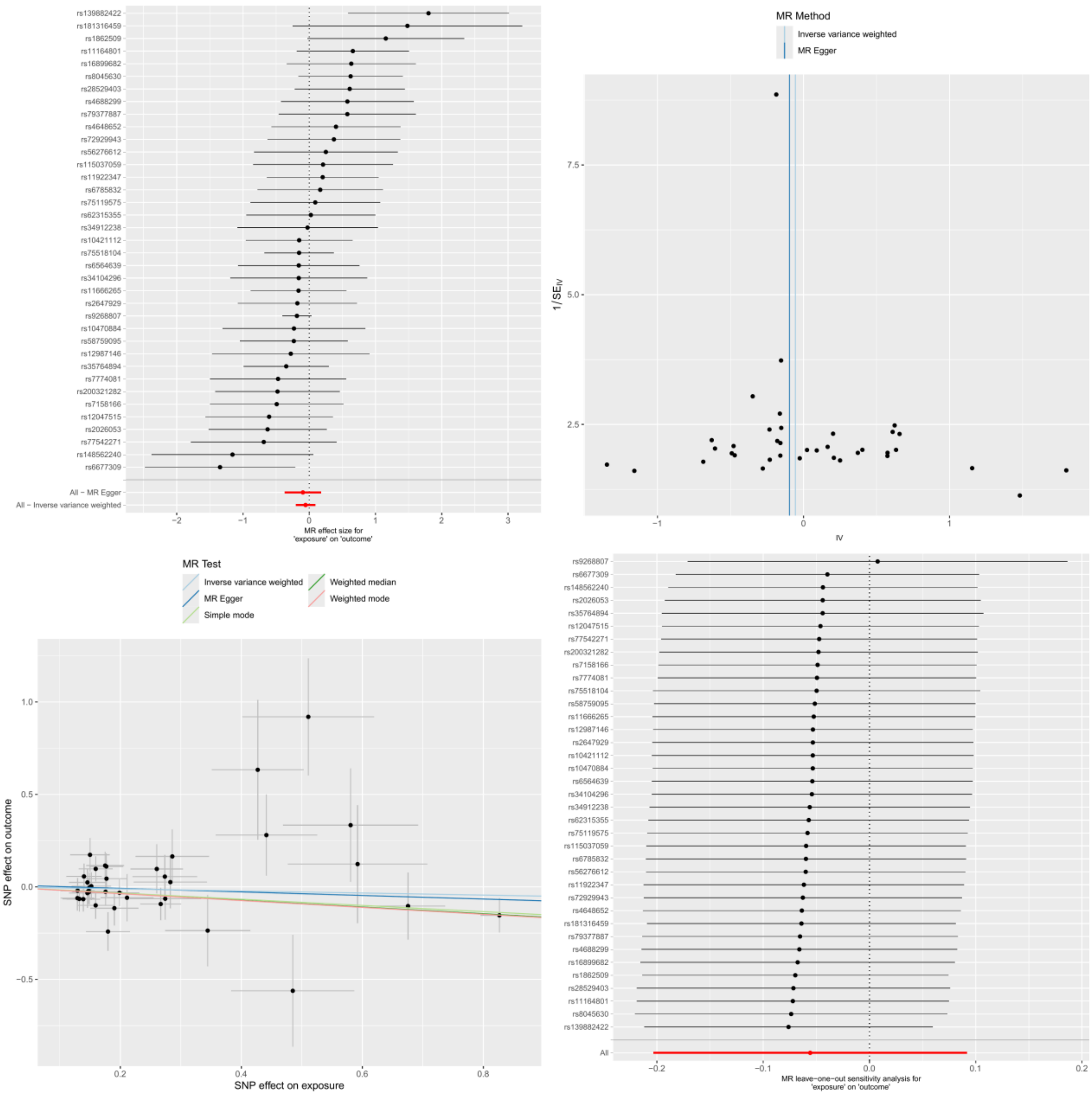

Supplemental Digital Content Figure 17. MR estimate results of the effects of MS on EBV EA-D antibody levels.

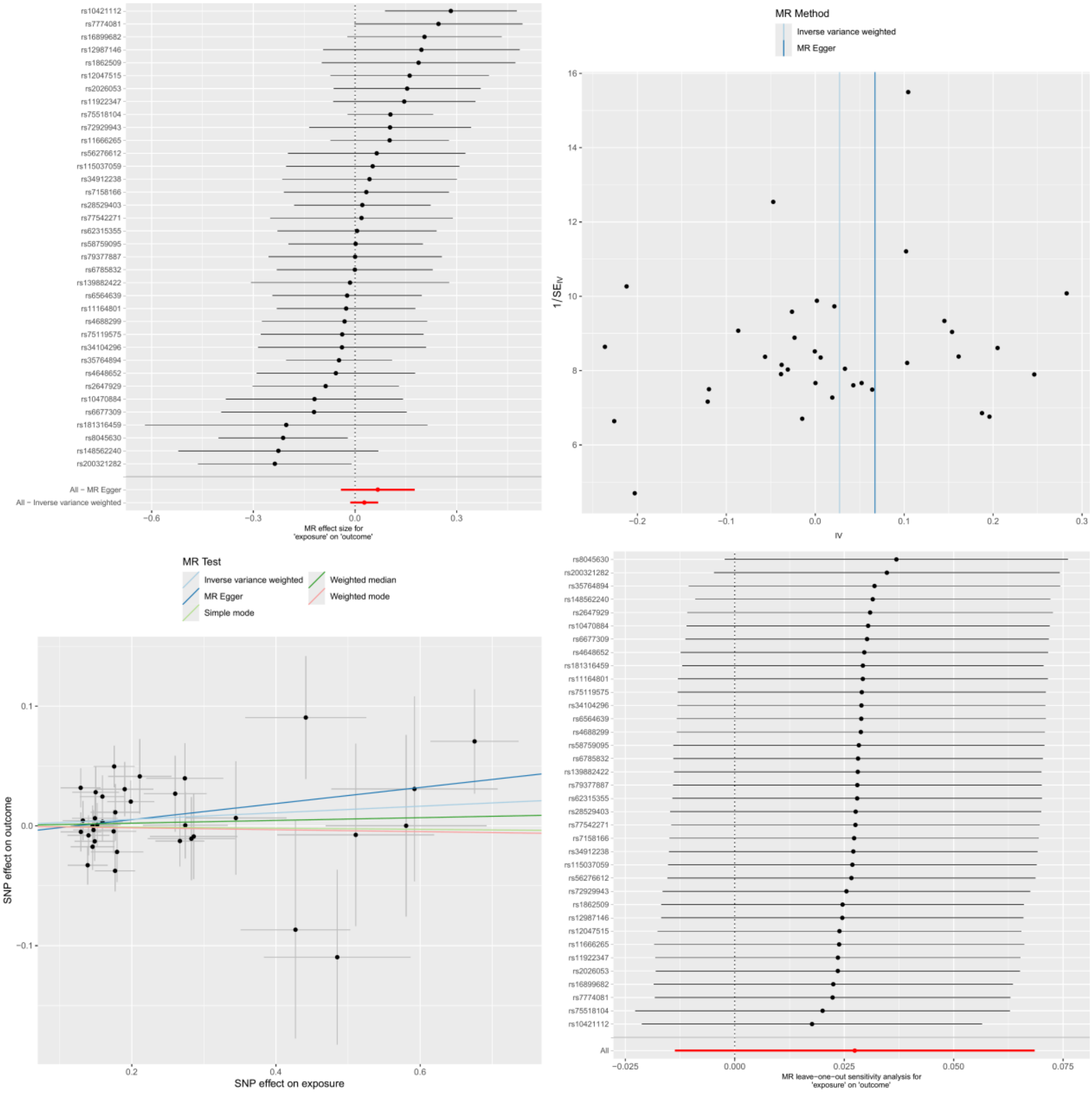

Supplemental Digital Content Figure 18. MR estimate results of the effects of MS on EBV EBNA-1 antibody levels.

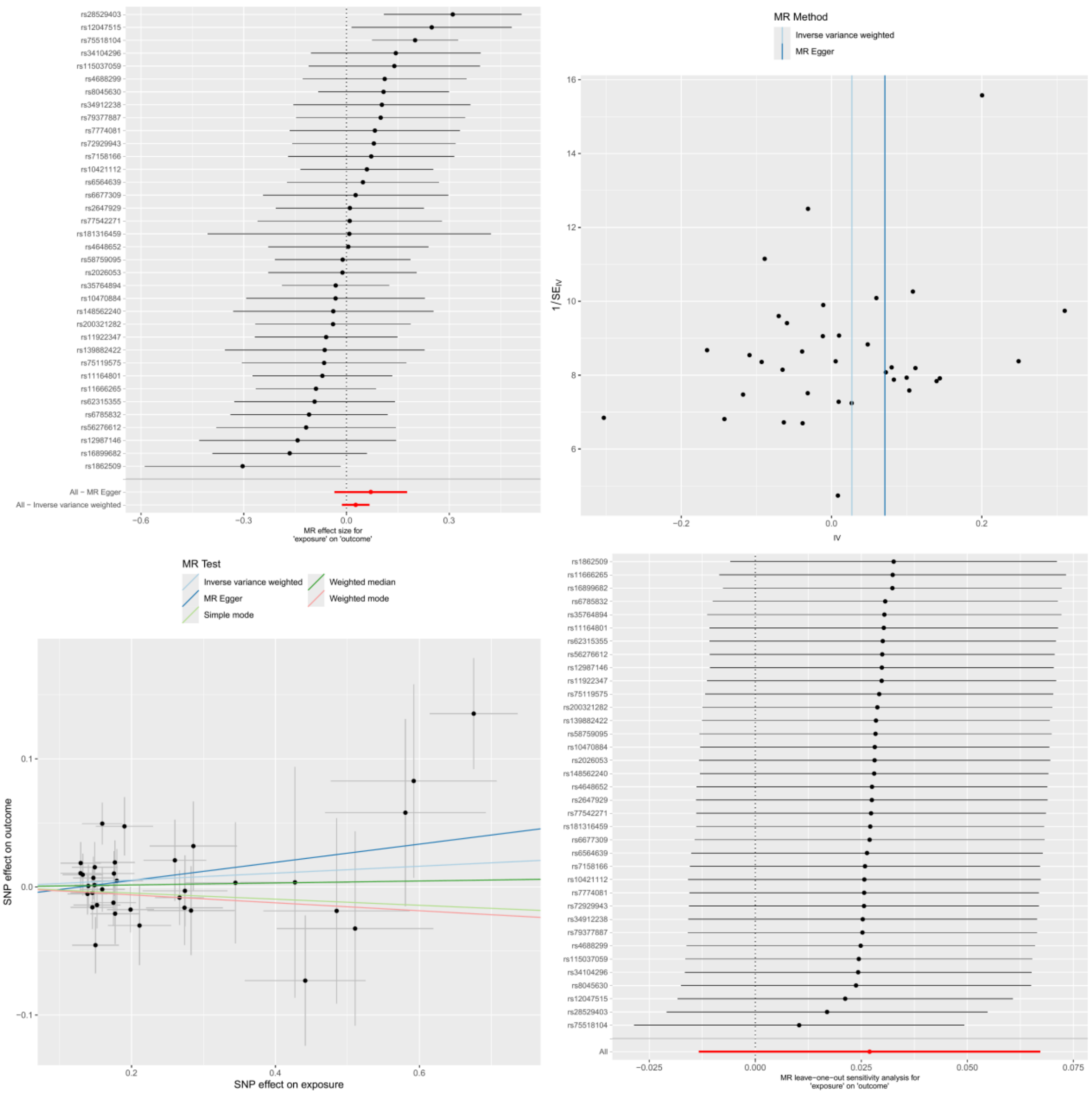

Supplemental Digital Content Figure 19. MR estimate results of the effects of MS on EBV VCA p18 antibody levels.

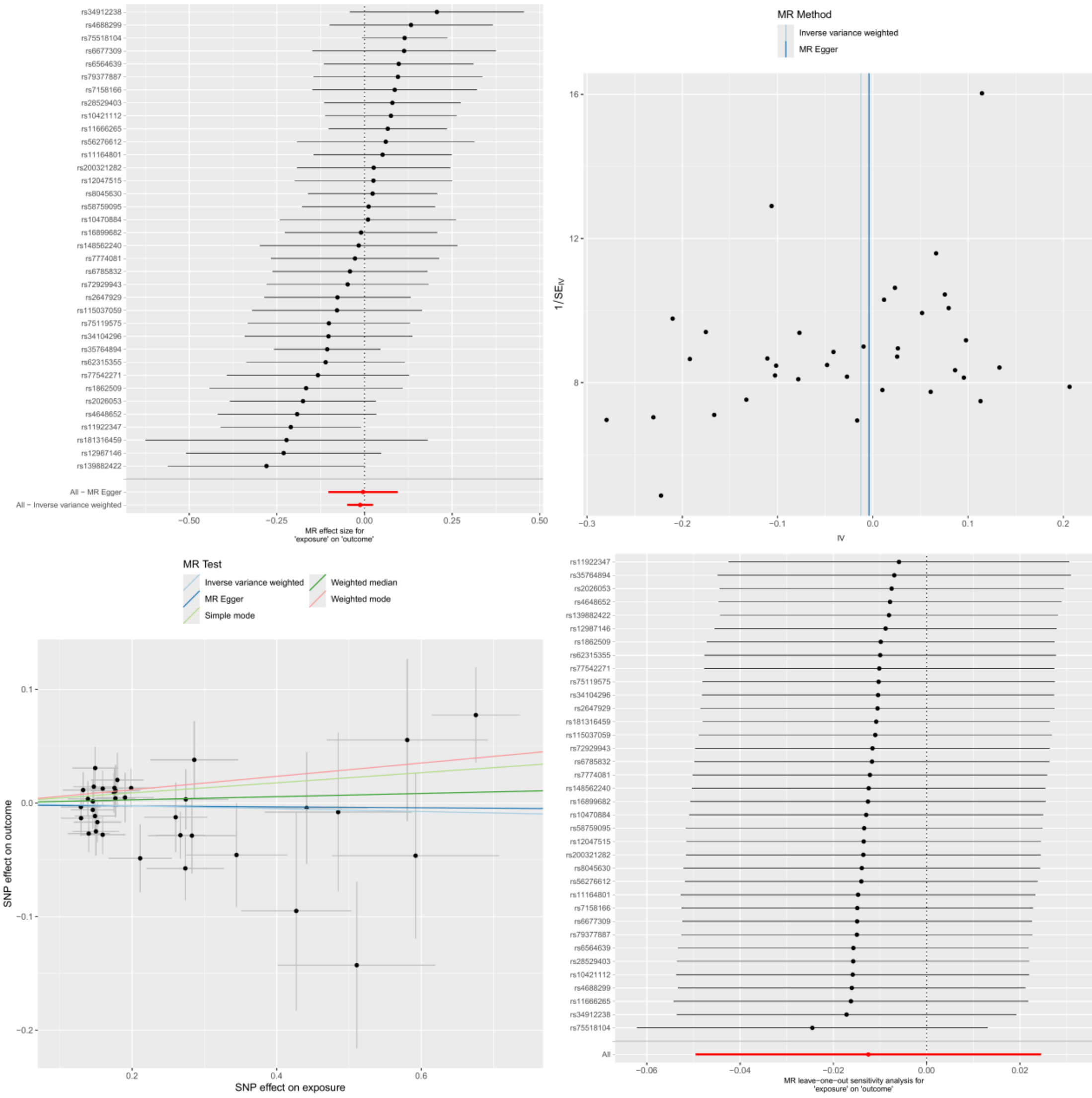

Supplemental Digital Content Figure 20. MR estimate results of the effects of MS on EBV ZEBRA antibody levels.

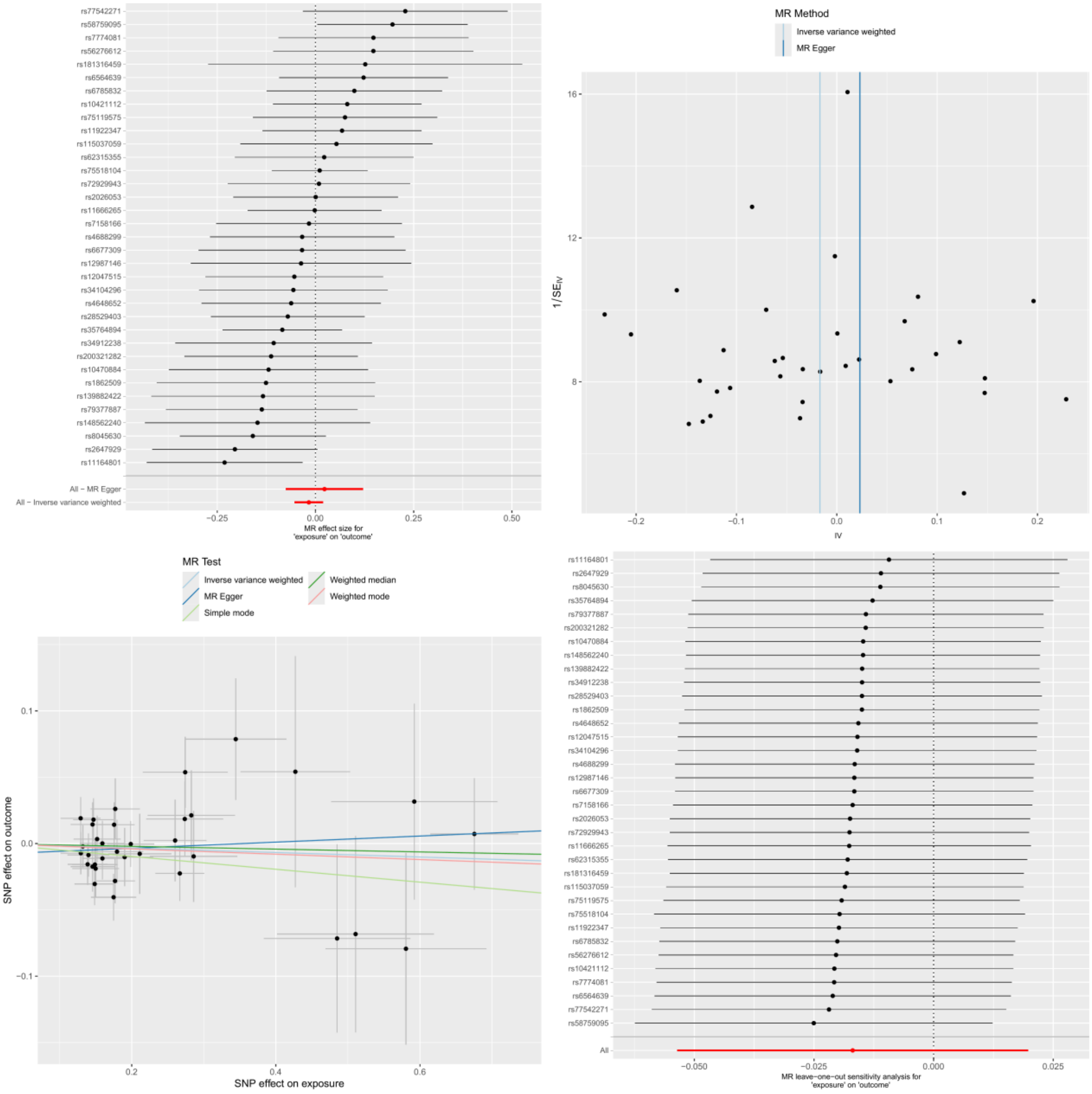

Supplemental Digital Content Figure 21. MR estimate results of the effects of NMO on Anti-EBV IgG seropositivity.

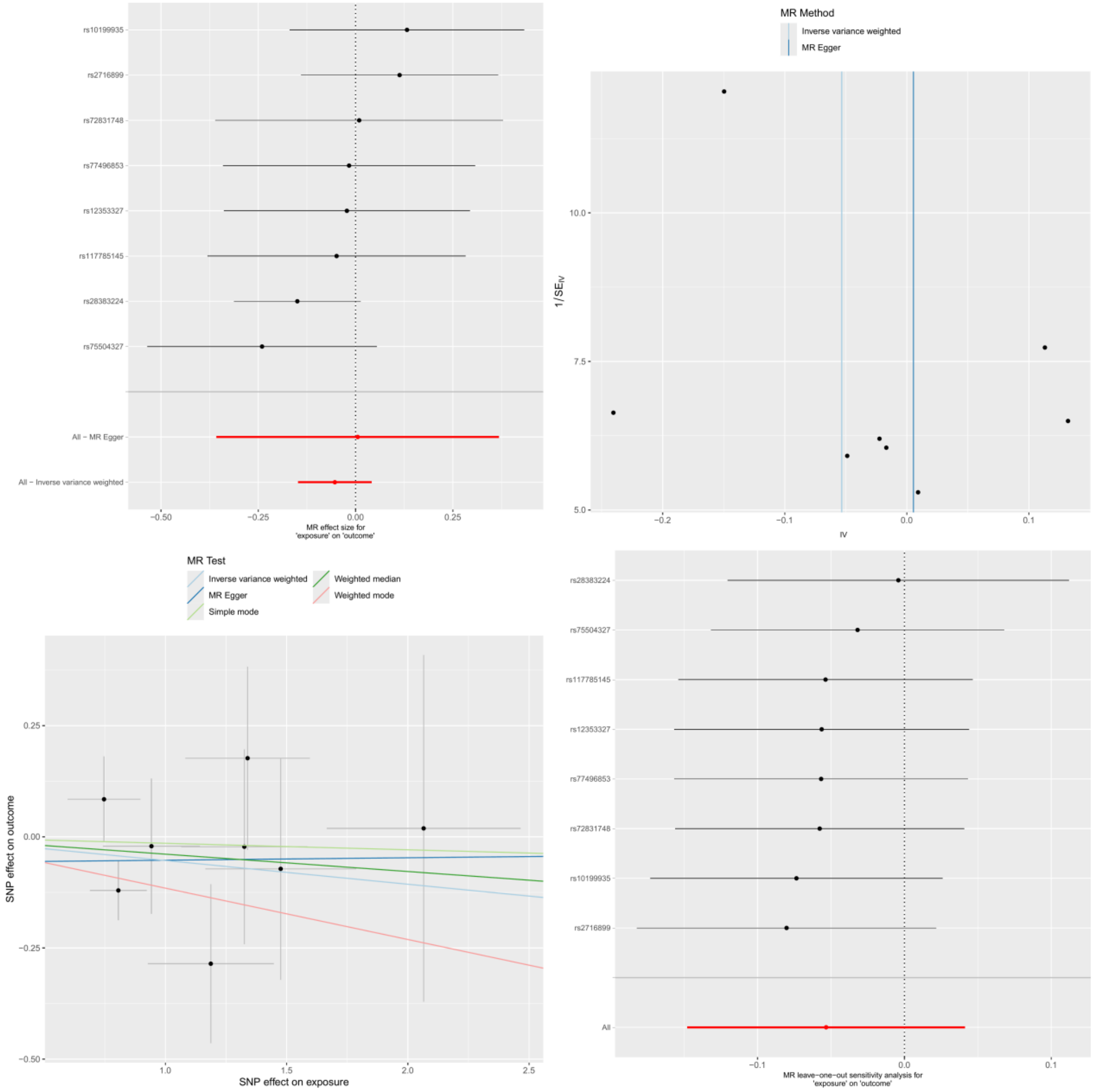

Supplemental Digital Content Figure 22. MR estimate results of the effects of NMO on EBV EA-D antibody levels.

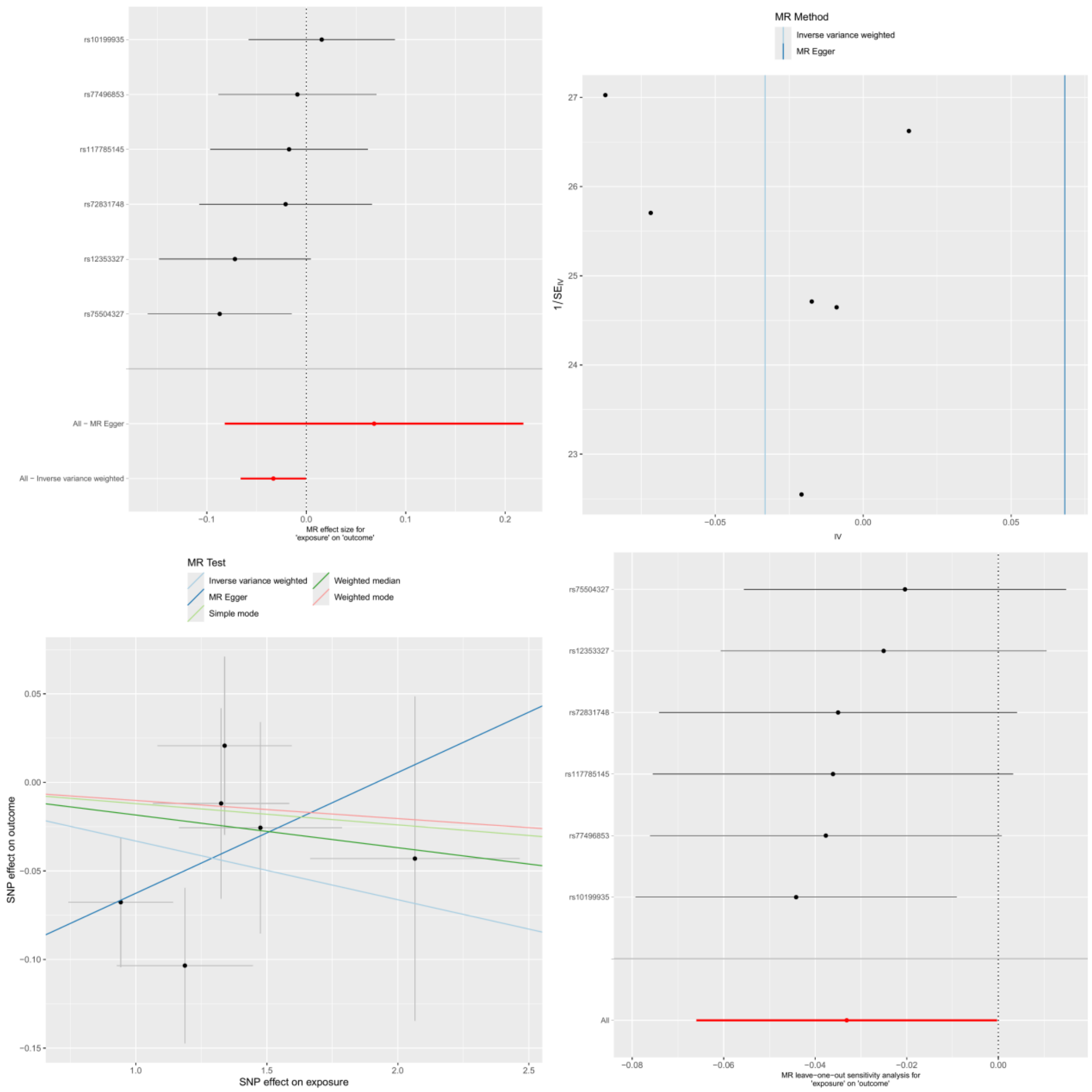

Supplemental Digital Content Figure 23. MR estimate results of the effects of NMO on EBV EBNA-1 antibody levels.

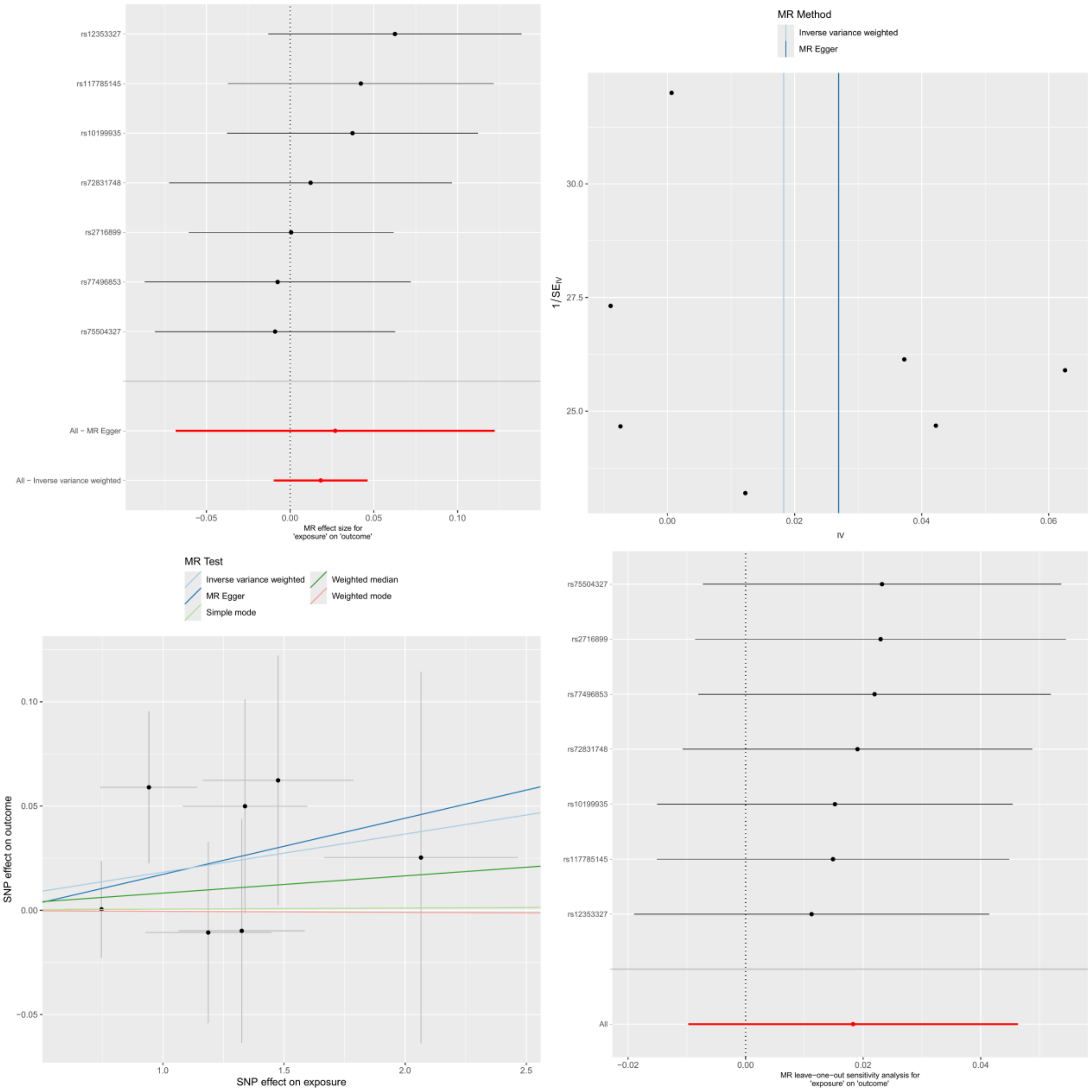

Supplemental Digital Content Figure 24. MR estimate results of the effects of NMO on EBV VCA p18 antibody levels.

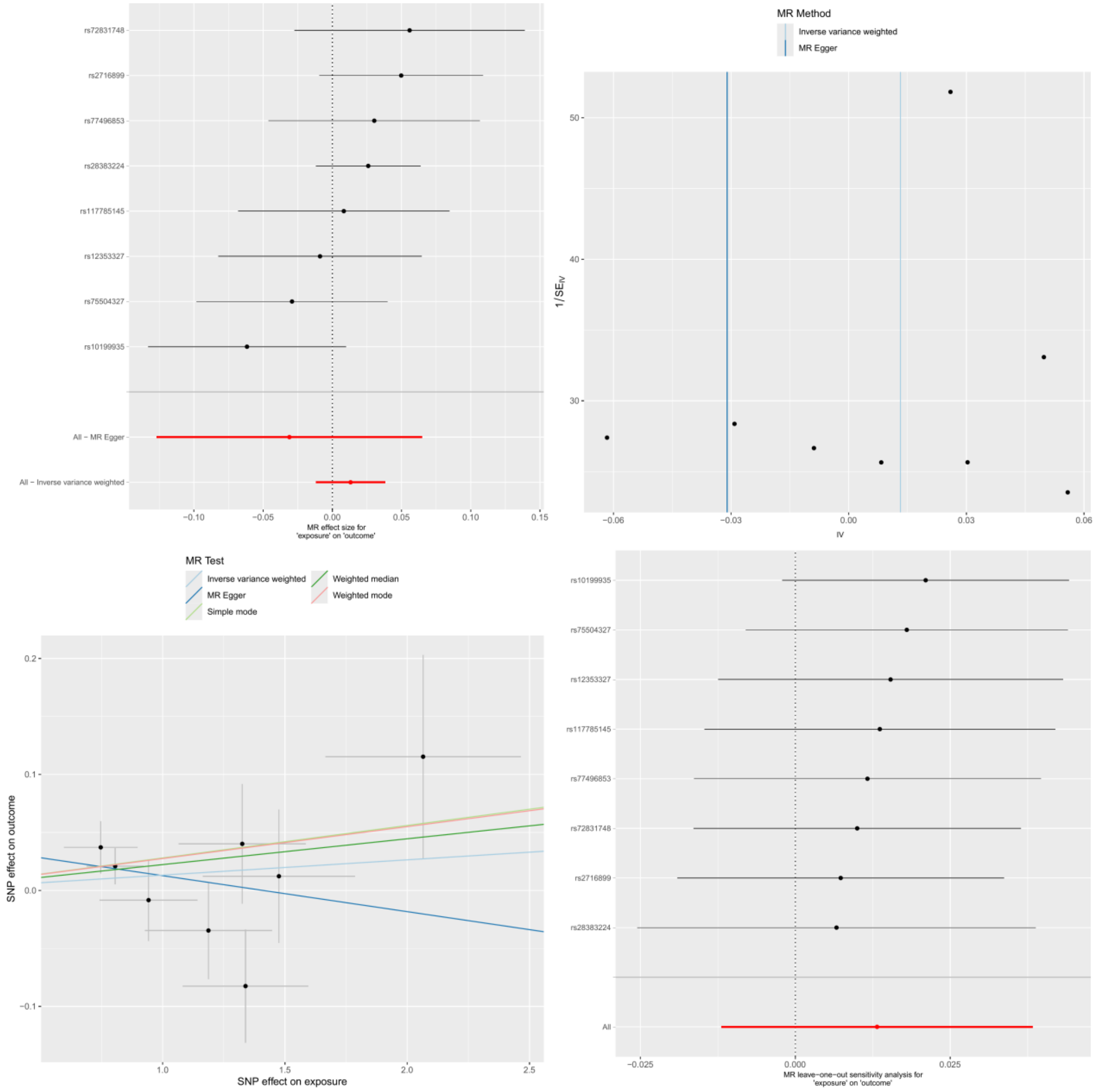

Supplemental Digital Content Figure 25. MR estimate results of the effects of NMO on EBV ZEBRA antibody levels.

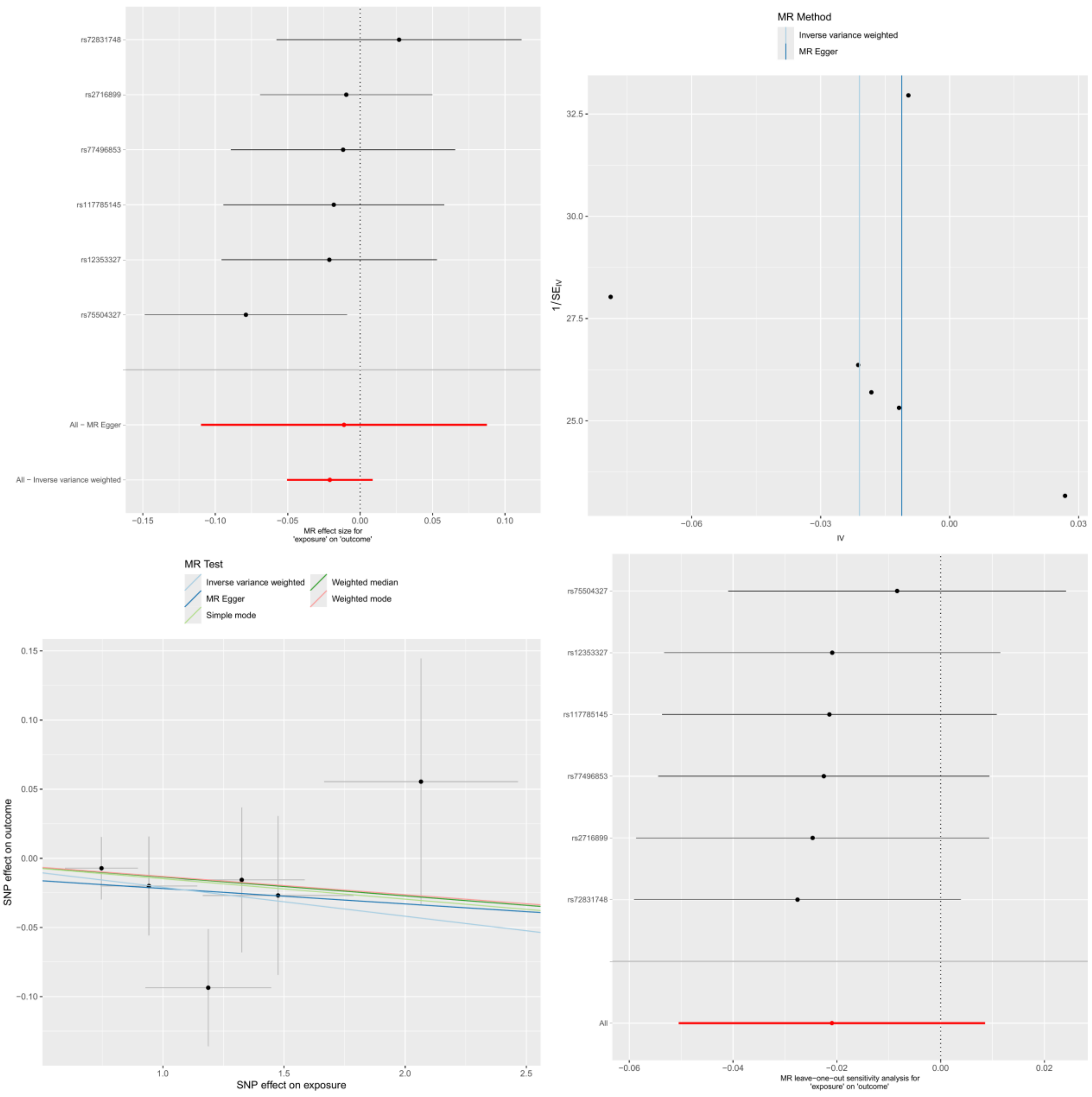

Supplemental Digital Content Figure 26. Causal effects of autoimmune neuroinflammatory diseases on Epstein-Barr virus antibodies: GSMR analysis with CoJo-based instrument selection and Steiger directionality validation.

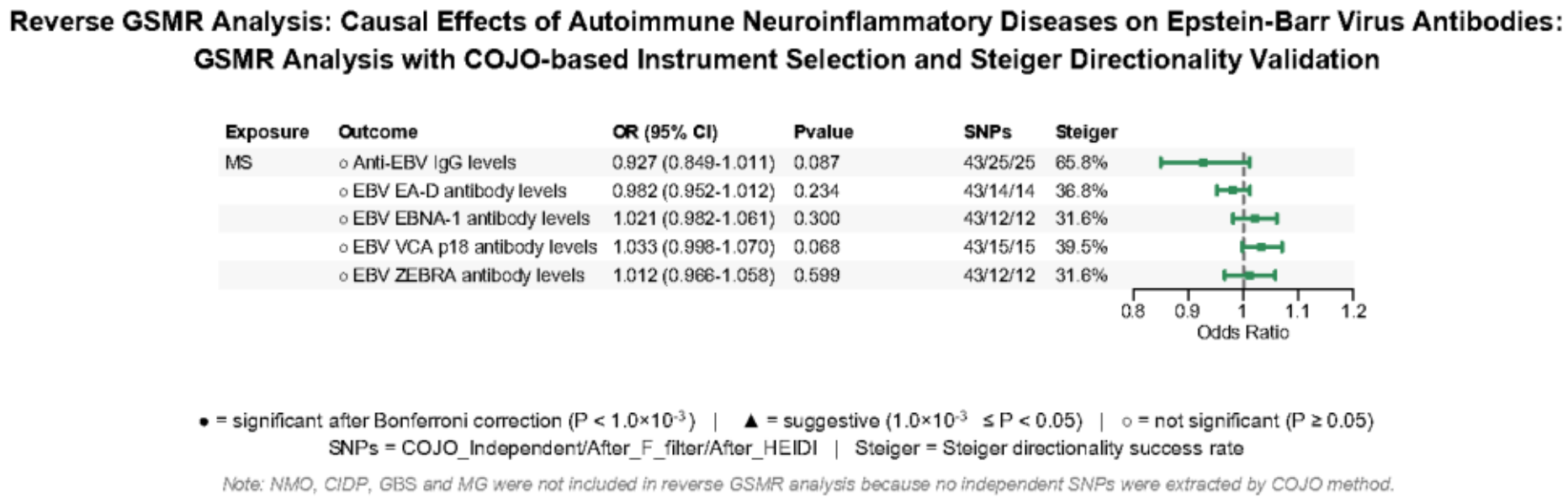

Supplement: Supplementary file 2 [file medi-105-e48264-s003.pdf]
